# Geographic distribution, clinical epidemiology and genetic diversity of the human oncogenic retrovirus HTLV-1 in Africa, the world’s largest endemic area

**DOI:** 10.3389/fimmu.2023.1043600

**Published:** 2023-02-03

**Authors:** Antoine Gessain, Jill-Léa Ramassamy, Philippe V. Afonso, Olivier Cassar

**Affiliations:** Institut Pasteur, Université Paris Cité, CNRS UMR 3569, Unité d’Épidémiologie et Physiopathologie des Virus Oncogènes, Paris, France

**Keywords:** HTLV-1, Africa, epidemiology, genetic variability, ATL, HAM/TSP

## Abstract

The African continent is considered the largest high endemic area for the oncogenic retrovirus HTLV-1 with an estimated two to five million infected individuals. However, data on epidemiological aspects, in particular prevalence, risk factors and geographical distribution, are still very limited for many regions: on the one hand, few large-scale and representative studies have been performed and, on the other hand, many studies do not include confirmatory tests, resulting in indeterminate serological results, and a likely overestimation of HTLV-1 seroprevalence. For this review, we included the most robust studies published since 1984 on the prevalence of HTLV-1 and the two major diseases associated with this infection in people living in Africa and the Indian Ocean islands: adult T-cell leukemia (ATL) and tropical spastic paraparesis or HTLV-1-associated myelopathy (HAM/TSP). We also considered most of the book chapters and abstracts published at the 20 international conferences on HTLV and related viruses held since 1985, as well as the results of recent meta-analyses regarding the status of HTLV-1 in West and sub-Saharan Africa. Based on this bibliography, it appears that HTLV-1 distribution is very heterogeneous in Africa: The highest prevalences of HTLV-1 are reported in western, central and southern Africa, while eastern and northern Africa show lower prevalences. In highly endemic areas, the HTLV-1 prevalence in the adult population ranges from 0.3 to 3%, increases with age, and is highest among women. In rural areas of Gabon and the Democratic Republic of the Congo (DRC), HTLV-1 prevalence can reach up to 10-25% in elder women. HTLV-1-associated diseases in African patients have rarely been reported *in situ* on hospital wards, by local physicians. With the exception of the Republic of South Africa, DRC and Senegal, most reports on ATL and HAM/TSP in African patients have been published by European and American clinicians and involve immigrants or medical returnees to Europe (France and the UK) and the United States. There is clearly a huge underreporting of these diseases on the African continent. The genetic diversity of HTLV-1 is greatest in Africa, where six distinct genotypes (a, b, d, e, f, g) have been identified. The most frequent genotype in central Africa is genotype b. The other genotypes found in central Africa (d, e, f and g) are very rare. The vast majority of HTLV-1 strains from West and North Africa belong to genotype a, the so-called ‘Cosmopolitan’ genotype. These strains form five clades roughly reflecting the geographic origin of the infected individuals. We have recently shown that some of these clades are the result of recombination between a-WA and a-NA strains. Almost all sequences from southern Africa belong to Transcontinental a-genotype subgroup.

## 1 Introduction

In 1984-1986, shortly after the discovery of the first oncogenic human retrovirus (HTLV-1) in 1980 (1), researchers mostly from Europe and North America, suggested that this virus might be endemic to certain regions of Africa (2–4). Almost 40 years later, the available data remain fragmentary for certain regions, but it is widely accepted that the African continent constitutes the largest endemic area for HTLV-1, with several million infected people (5). This review aims to provide as comprehensive and exhaustive as possible an overview of HTLV-1 infection and associated diseases in Africa and in Indian Ocean islands. Based on a rigorous analysis of published data and our expertise in the field, we provide the most probable figures for the prevalence, distribution and risk factors of HTLV-1 in different regions of Africa. We also describe what is currently known about HTLV-1-associated diseases, such as adult T-cell leukemia (ATL), HTLV-1 associated myelopathy (HAM/TSP), and infective dermatitis (ID), in Africa. Finally, we present data on the genetic diversity of HTLV-1 in Africa, focusing on our most recent data on recombinant strains.

In this analysis of the available data for HTLV-1 in Africa, we were confronted with seven critical issues:

1. Data are lacking for several regions. HTLV-1 has been little studied, if at all, in many African countries. This is the case, in particular, for some heavily populated regions of North and East Africa including the Horn of Africa. There is a critical need to perform studies in these areas.
2. The major epidemiological features of HTLV-1 infection include an increase in prevalence with age, and a higher prevalence in women than in men (6). An absence of data concerning the age and sex of the studied populations therefore precludes, in many cases, reliable comparisons of prevalence estimates.
3. A large proportion of the epidemiological studies on HTLV-1 performed in Africa was conducted on relatively small groups of people and focused on either blood donors or pregnant women. These two populations are not representative of the general population and are not comparable. Indeed, studies of the population of pregnant women provide information exclusively on the prevalence in infection with HTLV-1 in young women. Similarly, in West and Central Africa, blood donors are often young men, university students or soldiers, or, in some cases, relative of hospitalized patients requiring transfusions (7, 8). We were able to identify only a few studies that were truly representative of a country or rural/urban region, in Gabon and Guinea-Bissau for example. More studies on a more representative general population are required, particularly in the most populous countries of Africa.
4. HTLV-1-associated diseases in African patients have rarely been reported *in situ*, in hospital wards, by local physicians. With the exception of the Republic of South Africa, the Democratic Republic of the Congo (DRC) and Senegal, most reports on ATL and HAM/TSP in African patients have been published by European and American clinicians and relate to immigrants or medical returnees in Europe (France and the UK) and the USA. There are many reasons for local underreporting of ATL: a lack of awareness of these rare clinical entities among most clinicians, including many hematologists, the rapid progression observed in the absence of effective treatment, a lack of availability of the diagnostic tests for HTLV-1, immunostaining for pathology purposes (CD4, CD25), and tests for molecular T-cell clonality. The clinical and epidemiological features of ATL, HAM/TSP and ID therefore remain virtually unknown in many parts of Africa.
5. Most epidemiological studies on HTLV-1 are based on serological methods, which can be difficult to interpret in samples from sub-Saharan Africans. Indeed, in many tropical regions, particularly in sub-Saharan Africa, many ELISA-positive results have been found to be false positives. Epidemiological studies based on ELISA alone therefore overestimate the prevalence of HTLV-1, by a factor two to six, depending on the study. Historical publications (1984–1989) were valuable, as they drew attention to the high prevalence of HTLV-1 in certain regions/countries of Africa, but in most of the cases they largely overestimated HTLV-1 seroprevalences, and cannot, therefore, be included in a rigorous epidemiological review (2). Thus, an initial screening test (usually an ELISA or an equivalent method) should ideally be performed, with or without replication, and then a second test based on a different method should be used to confirm the initial results. The current reference tests for such validation are western blotting or the Innogenetics line immunoassay (INNO-LIA) (9). Unfortunately, many recent studies have still disregarded this problem and were based on a single serological detection technique.
6. Indeterminate seroreactivity patterns, with partial immunoreactivity against HTLV-1 on Western blots (therefore not corresponding to the defining criteria of the confirmatory kits), are often observed with samples from Central Africa. In a few cases, these indeterminate patterns correspond to individuals infected with defective HTLV-1 viruses or with a low proviral load. These individuals are unlikely to transmit the virus, and the importance of their detection in terms of public health is debatable. In some cases, partial immunoreactivities may correspond to ongoing seroconversion in the individual concerned, as demonstrated by some longitudinal serological studies (10). Indeterminate profiles may indicate infection with related viruses, such as HTLV-2, which is mostly detected in the Pygmy populations of Cameroon (11) and the Democratic Republic of the Congo (DRC) (12), or HTLV-3 or -4, which are quite rare, with fewer than 10 cases reported in Central Africa (Cameroon and Gabon) (13–15). In most cases, these indeterminate patterns are not related to HTLV-1 infection.

Two indeterminate patterns on Western blotting, named HGIP (for HTLV-1 gag indeterminate patterns) or N (for New) profiles, have been shown to be frequent in Central Africa (16–18). They often result from cross-reactivity with other microbial agents, such as *Plasmodium falciparum* in Cameroon (16, 18). In populations displaying such patterns, the epidemiological features differ from those of HTLV-1 infection: there is no increase in prevalence with age, no female preponderance, no evidence of mother-to-child transmission and these patterns are not present in patients with HTLV-1-associated diseases (19).

7. Small clusters of high HTLV-1 prevalence are often observed, with a lower prevalence of the virus in neighboring regions. This has been clearly shown in southern Japan (20), some regions of South America (e.g. Argentina and French Guiana) (21) and in the Middle East, in the Mashhad region of Iran (22), for example. With this “microepidemiology”, it is difficult to estimate the prevalence within a given region accurately. This difficulty can be overcome only by performing large studies or combining many studies, to obtain a reliable figure representative of the region. Unfortunately, very few of the studies conducted in Africa have adopted such approaches.

## 2 Epidemiological aspects of HTLV-1 in Africa

### 2.1 Methods: Search strategy and data extraction for HTLV-1 infection, ATL and HAM/TSP cases in African countries and Indian Ocean Islands

We searched PubMed for studies published since 1983 on HTLV-1 prevalence and the two main diseases associated with HTLV-1 infection found in individuals of African origin: adult T-cell leukemia and tropical spastic paraparesis or HTLV-1-associated myelopathy. We also included most of the book chapters and abstracts published from 20 international conferences on HTLV and related viruses held since 1985. The results of recently published meta-analyses regarding the situation of HTLV-1 worldwide and in particular in West and sub-Saharan Africa, have also made it possible to find some original articles that have been used in this study (23–28).

Keyword searches were also performed to identify articles from gray literature sources, including Google Scholar and Web of Science. Restrictions were imposed regarding language (English, Spanish and French) and study design (cases of HTLV-1 infection confirmed by serological (Western blotting) or molecular (PCR) tests).

Medical Subject Heading (MeSH) terms and keyword searches were performed on PubMed and other databases (Google Scholar). Additional studies were identified through manual searches of the references of relevant studies and reviews. The search terms were combined with ‘and’ statements and searches were performed on article titles, abstracts and subjects.

The PubMed search terms used were: “HTLV-1” OR “HTLV1” OR “Human T-cell Lymphotropic Virus” OR “Human T-cell Lymphotropic Virus Type 1” OR “Human T-leukemia Virus Type 1” AND “ATL” OR “ATLL” OR “Adult T-cell leukemia” OR “Adult T-cell leukemia/lymphoma” OR “leukemia-lymphoma” OR “HAM/TSP” OR “Tropical spastic paraparesis” OR “HTLV-1-associated myelopathy” OR “Tropical spastic paraparesis/HTLV-1 associated myelopathy” OR “Infective dermatitis”. All these query items were associated with the name of a country or region in Africa (North, West, Central, East, South) and territories of the Indian Ocean region (Reunion Island, Republic of Seychelles, Madagascar and the Comoros). The former names of countries were also used (Upper Volta, Zaire and Swaziland).

The database searches were updated regularly, with the last update on August 3, 2022.

The titles and abstracts of the publications identified were screened independently by three reviewers (JL, AG and OC), with any publication deemed potentially relevant by either reviewer carried forward for full-text evaluation. Disagreements during full-text review were resolved by consensus.

### 2.2 Results

**Figure 1:**
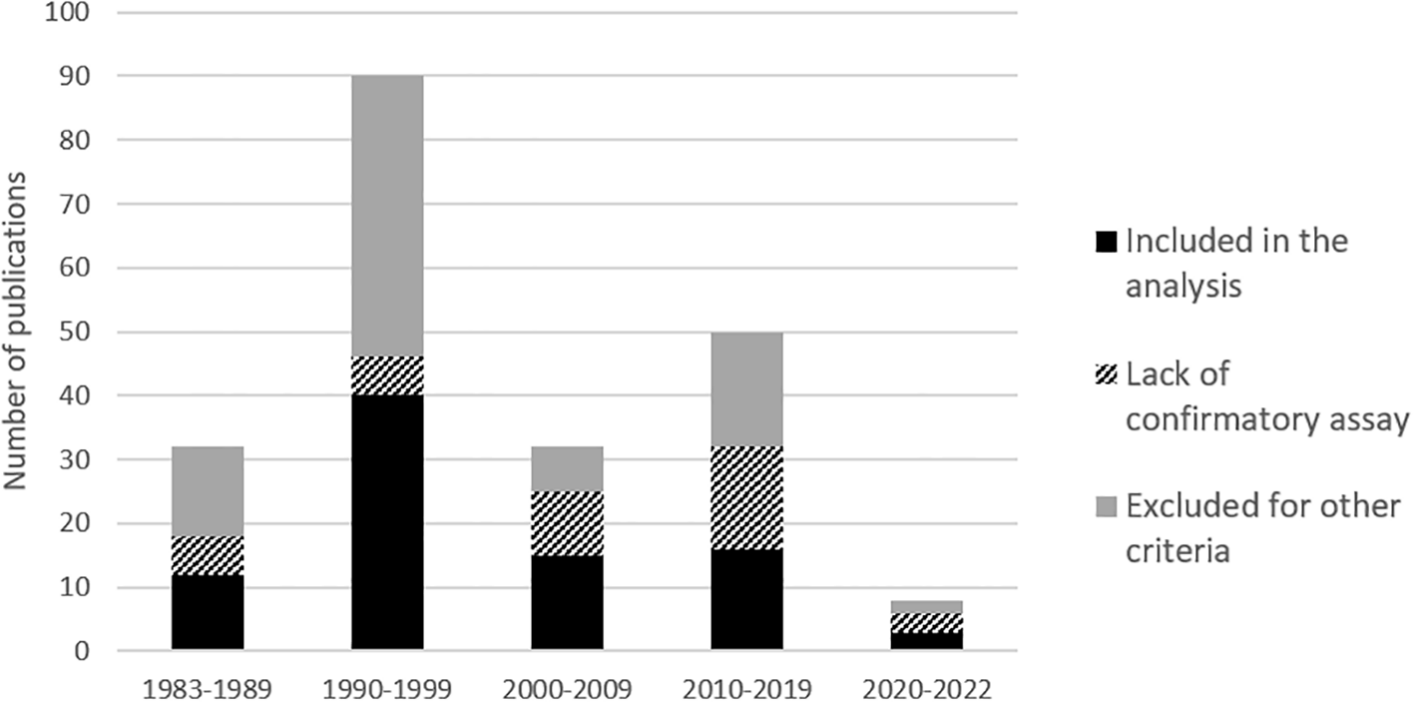
Histogram of number of publications per decades on ‘HTLV-1 prevalences’ in Africa, from 1983 to July 2022. The shades of gray to black represents the percentage of studies lacking confirmatory results and of studies excluded for other criteria that are detailed in figure 2 (mainly other type of population, low numbers and disease report studies).

**Figure 2:**
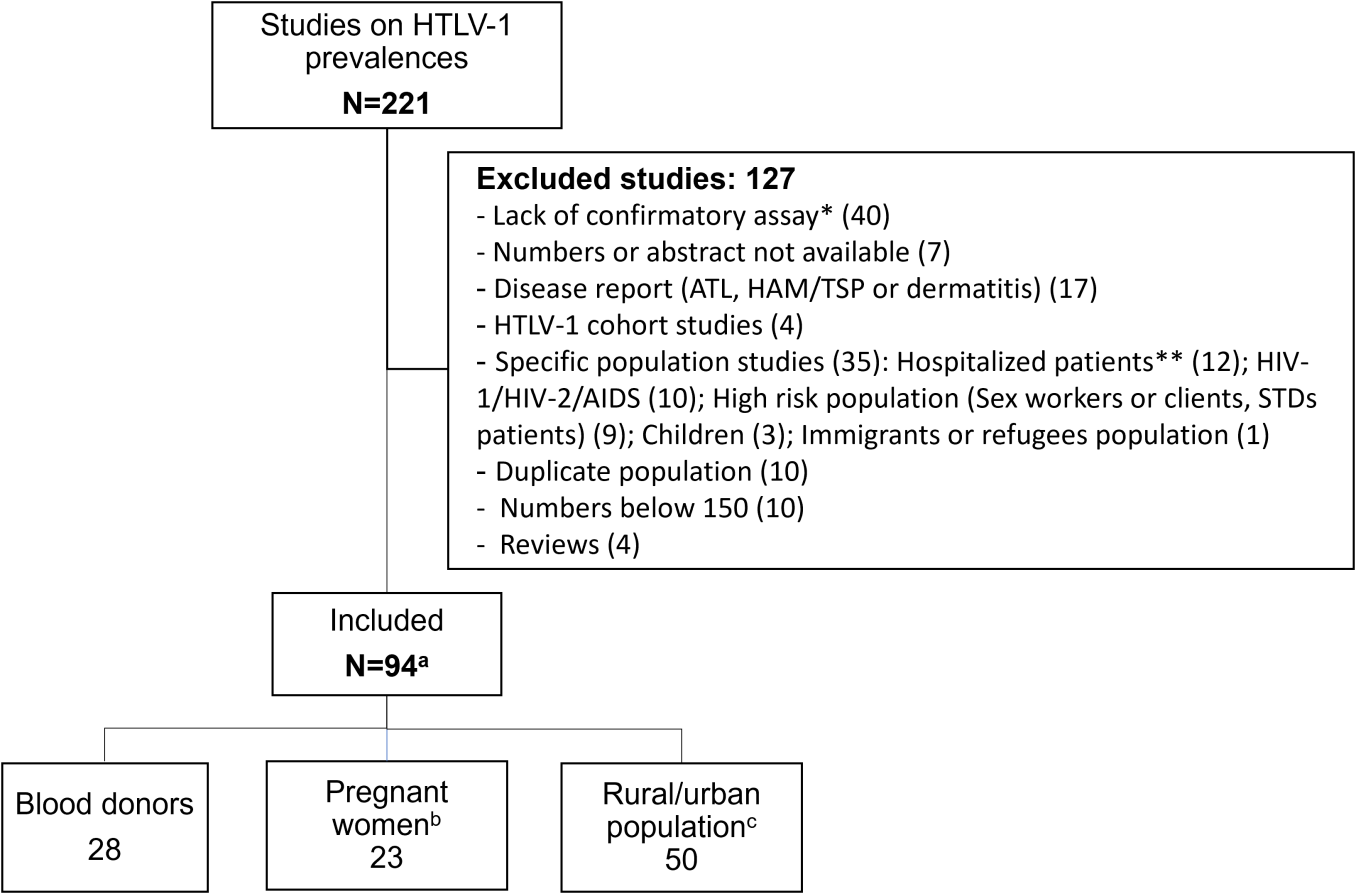
Flow chart of manuscript screened for prevalence of HTLV-1 in blood donors, pregnant women and rural or urban adult population, living in Africa. * Two studies lacking confirmatory assay but showing 0% HTLV-1 seroprevalence with ELISA were included and were not counted in this line. ** Hospitalized patients included neurological, hematological, malignant, Kaposi, Leprosy patients and other. **(a)** Numbers in each study population category do not add up as some studies had data on different populations (blood donors, pregnant women and urban or rural adult population). These studies were counted in each category. **(b)** Two studies on healthy mothers were included. **(c)** Two studies on rural out-patients, presenting no severe pathology were included and considered as rural population.

We identified 220 references relating to epidemiological studies on HTLV-1 performed on the African continent. Most were published in the 1990s (**Figure 1**; **Table S1**). As indicated above (see Introduction, point 5), one major limitation of several of these studies was the absence of serological confirmation assays. As explained above, high levels of cross-reactivity with other pathogens can result in a false positives and consequent overestimate, hindering the interpretation of single ELISA results (**Figure 2**; **Table S1**). Thus, in principle, only studies on large populations (more than 150 people) and including serological confirmation assays (immunofluorescence IFA, immunoprecipitation (RIPA), Western blotting (WB), Innogenetics line immunoassay (INNO-LIA)) or molecular diagnosis (e.g. PCR), should be considered. However, we also chose to include studies in which no samples tested positive by ELISA, as there is no risk of overestimation. Studies on populations at high risk of HTLV-1 infection, such as HIV-infected individuals, sex workers or patients with other STIs, were excluded, together with studies on hospitalized patients (**Figure 2**). Based on these criteria, 94 studies focusing on the prevalence of HTLV-1 in rural or urban adult populations, blood donors or pregnant women were included in the analyses (**Figure 2**).

**Figure 3:**
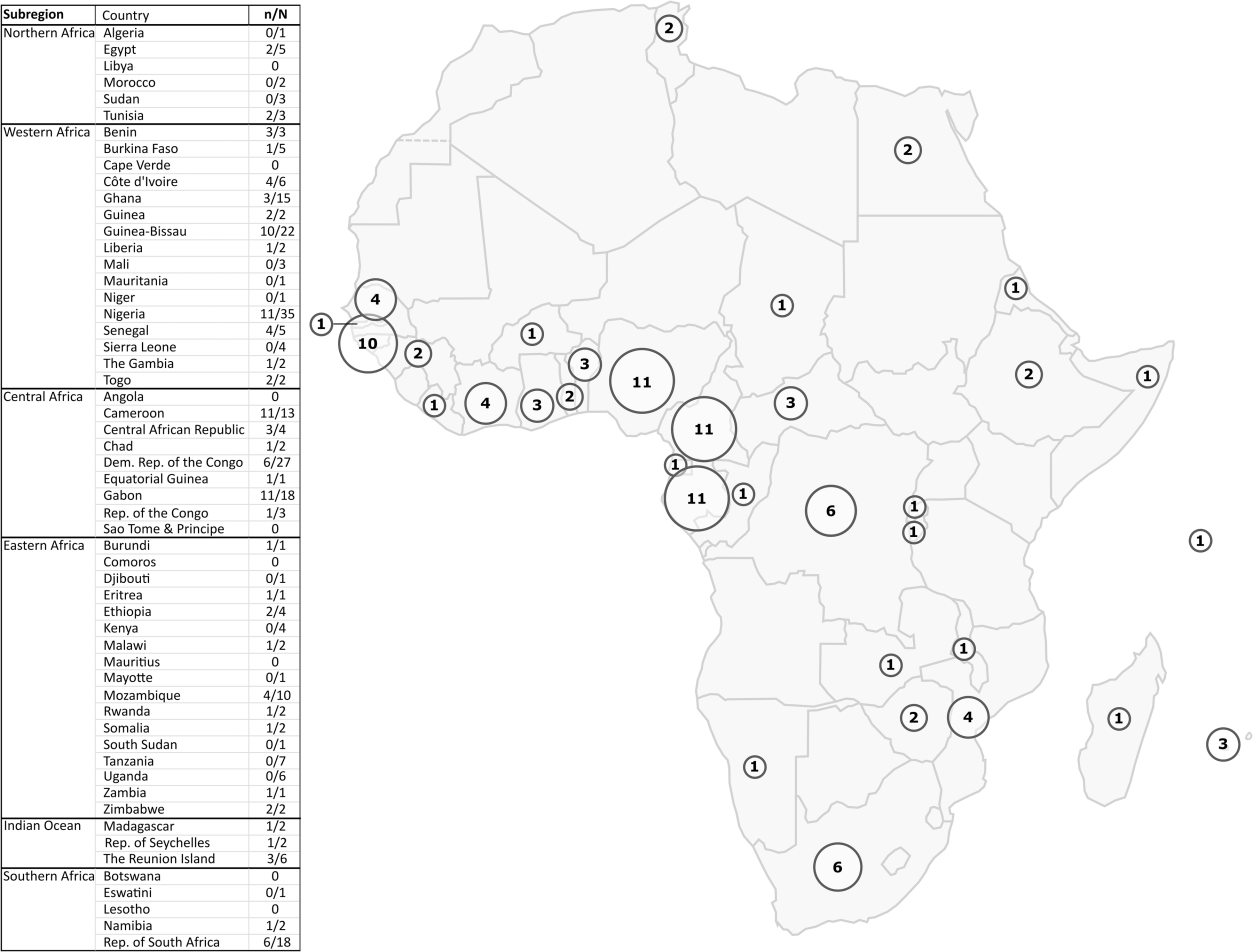
Map of Africa showing the geographic location of HTLV-1 prevalence studies. The circles represent the number of HTLV-1 prevalence studies among blood donors, pregnant women, or the adult rural or urban population in each country that were included in the representation of results. For each country, the n/N column of the table indicates the number of included studies out of the total number of HTLV-1 epidemiological studies concerning that country. Studies with data on different countries were counted for each concerned country (i) both in the number of included studies (ni) and in the total number of studies (Ni). As a result, the sum of ni for all countries is greater than the total number of studies included in our study (n). The same is true for the total number of studies. The exclusion criteria are detailed in **Figure 2**.

The vast majority of the studies were carried out in Central and West Africa, leaving much of North and East Africa with little information available on the presence of HTLV-1 (**Figure 3**).

#### 2.2.1 Pregnant women

**Figure 4:**
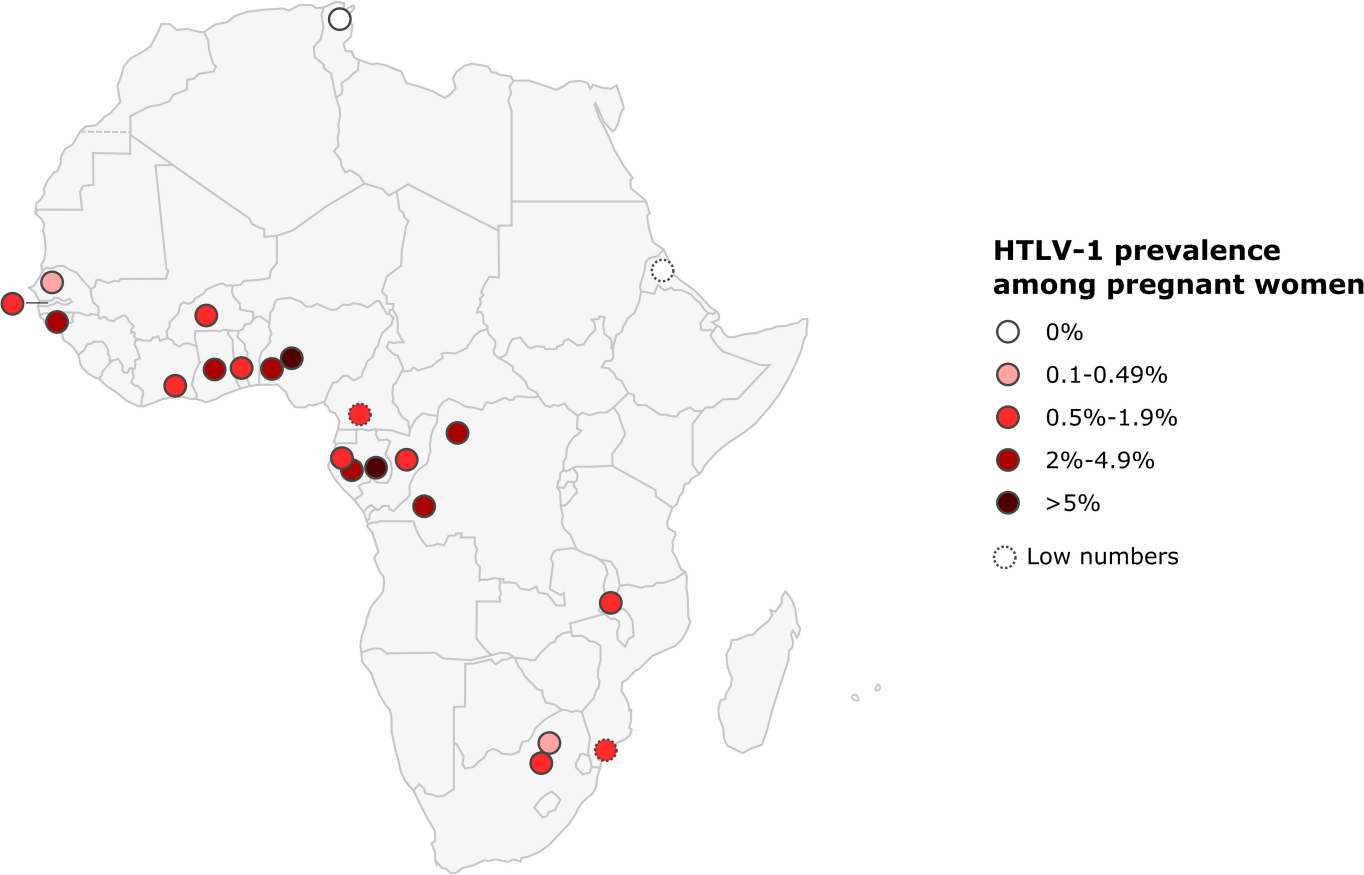
Map of Africa showing location and results of HTLV-1 prevalence studies among pregnant women. The range of HTLV-1 prevalence is indicated by color of red gradient. In case of great geographical or inter-studies variations in prevalence within a country, each result was represented separately at the site of the study location (when available). Studies with low numbers (below 150) are indicated with a dashed line.

The prevalence of HTLV-1 in pregnant women ranged from 0.2 to 7.7% in West Africa, 0.6-6.8% in Central Africa, 0% to 1% in East Africa, and 0.2 to 0.6% in Southern Africa. No HTLV-1 infection was detected in the only study performed in North Africa. The highest prevalences were reported in Gabon (2.1-6.8%), DRC (5%), Nigeria (3.3-7.7%), and Guinea-Bissau (2.2-3.3%) (**Figure 4**; **Table 1**).

**Table 1:** HTLV-1 prevalence among African pregnant women.

Subregion	Country	HTLV-1 prevalences in PW	Total nb of PW	References
Northern Africa	Tunisia	0%*	442	(29)
Western Africa	Burkina Faso	0.8%	492	(30)
	Côte d’Ivoire	1.8-1.9%	1327	(31, 32)
	The Gambia	1.20%	909	(33)
	Ghana	2-2.1%	2080	(34, 35)
	Guinea-Bissau	2.2-3.3%	1503	(36, 37)
	Nigeria	3.3-7.7%	824	(38, 39)
	Senegal	0.2-0.4%	696	(29, 32)
	Togo	1.20%	565	(32)
Central Africa	Cameroon	0.60%	170	(40)
	Rep. of the Congo	0.70%	2070	(41)
	DR. of the Congo	3.7-4.6%	1574	(42, 43)
	Gabon	2.1-6.8%	2191	(44–46)
Eastern Africa	Eritrea	0%	113	(47)
	Malawi	1.00%	418**	(48)
	Mozambique	0.70%	132	(49)
Southern Africa	Rep. of South Africa	0.2-0.56%	1687	(43, 50)

DR, Democratic Republic; PW, pregnant women; Rep, Republic; * ELISA only; ** mothers.

#### 2.2.2 Blood donors

**Figure 5:**
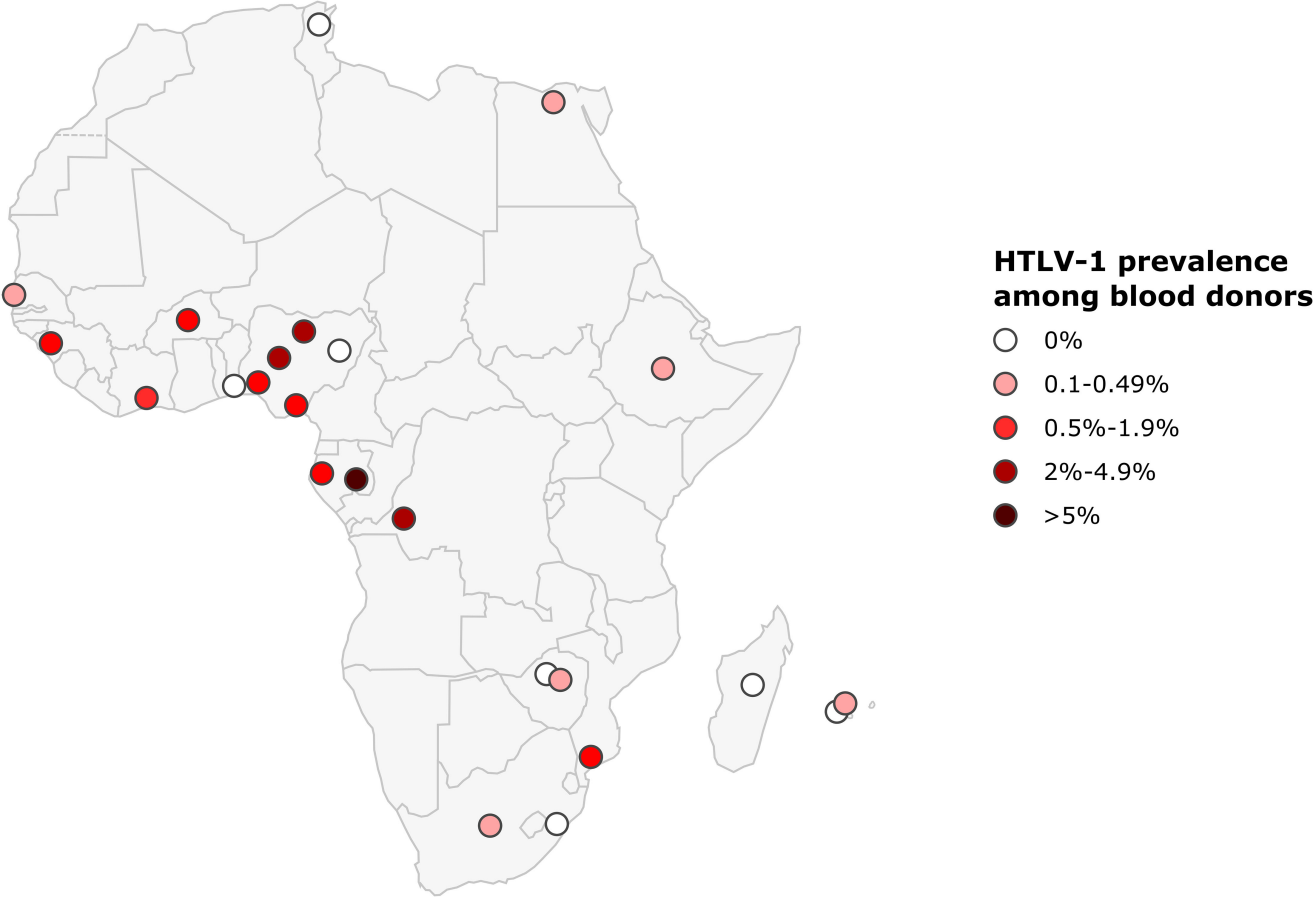
Map of Africa showing location and results of HTLV-1 prevalence studies among blood donors. The range of HTLV-1 prevalence is indicated by color of red gradient. In case of great geographical or inter-studies variations in prevalence within a country, each result was represented separately at the site of the study location (when available).

The prevalence of HTLV-1 in blood donors ranged from 0 to 0.1% in North Africa, 0 to 2.6% in West Africa, 0.7 to 6% in Central Africa, 0 to 1.1% in East Africa, and 0 to 0.1% in Southern Africa. As for pregnant women, prevalences were highest in Central African countries, reaching 6% among blood donors from East Gabon, 4% in DRC, and in 2.6% in some Nigerian blood donors (West Africa) (2.6%) (**Figure 5**; **Table 2**).

**Table 2:** HTLV-1 prevalence among African blood donors.

Subregion	Country	HTLV-1 prevalence	N BD	References
Northern Africa	Egypt	0.12%	866	(51)
	Tunisia	0%	500	(52)
Western Africa	Benin	0%	1300	(53)
	Burkina Faso	1.0%	191	(30)
	Côte d’Ivoire	1.70%	414	(54)
	Guinea	1.20%	1785	(55)
	Nigeria	0-2.6%	1327	(4, 56–58)
	Senegal	0.16%	4900	(59)
Central Africa	Cameroon	0%	100	(40)
	DR. of the Congo	4%	530	(43)
	Gabon	0.74%-6%	3756	(8, 44)
Eastern Africa	Ethiopia	0.19%	1600	(60)
	Mozambique	0.89-1.1%	3597	(61, 62)
	Zimbabwe	0*-0.1%	1509	(63, 64)
Southern Africa	Rep. of South Africa	0-0.13%	85777	(65, 66)
Indian Ocean	Madagascar	0%	198	(29)
	The Reunion Island	0-0.03%	44158	(67–69)

N BD: total number of blood donors studied.

* ELISA only; DR. of the Congo, Democratic Republic of the Congo; Rep, Republic.

As indicated above (point 3), populations of blood donors are not representative of the general (urban or rural) population. In Gabon, the age- and sex-adjusted prevalence of HTLV-1 was four times higher in the rural population than in blood donors (8). However, studies of HTLV-1 infection in blood donors provide a useful indicator of the risk of transmission *via* contaminated blood (7).

#### 2.2.3 Adult rural and urban populations

**Figure 6:**
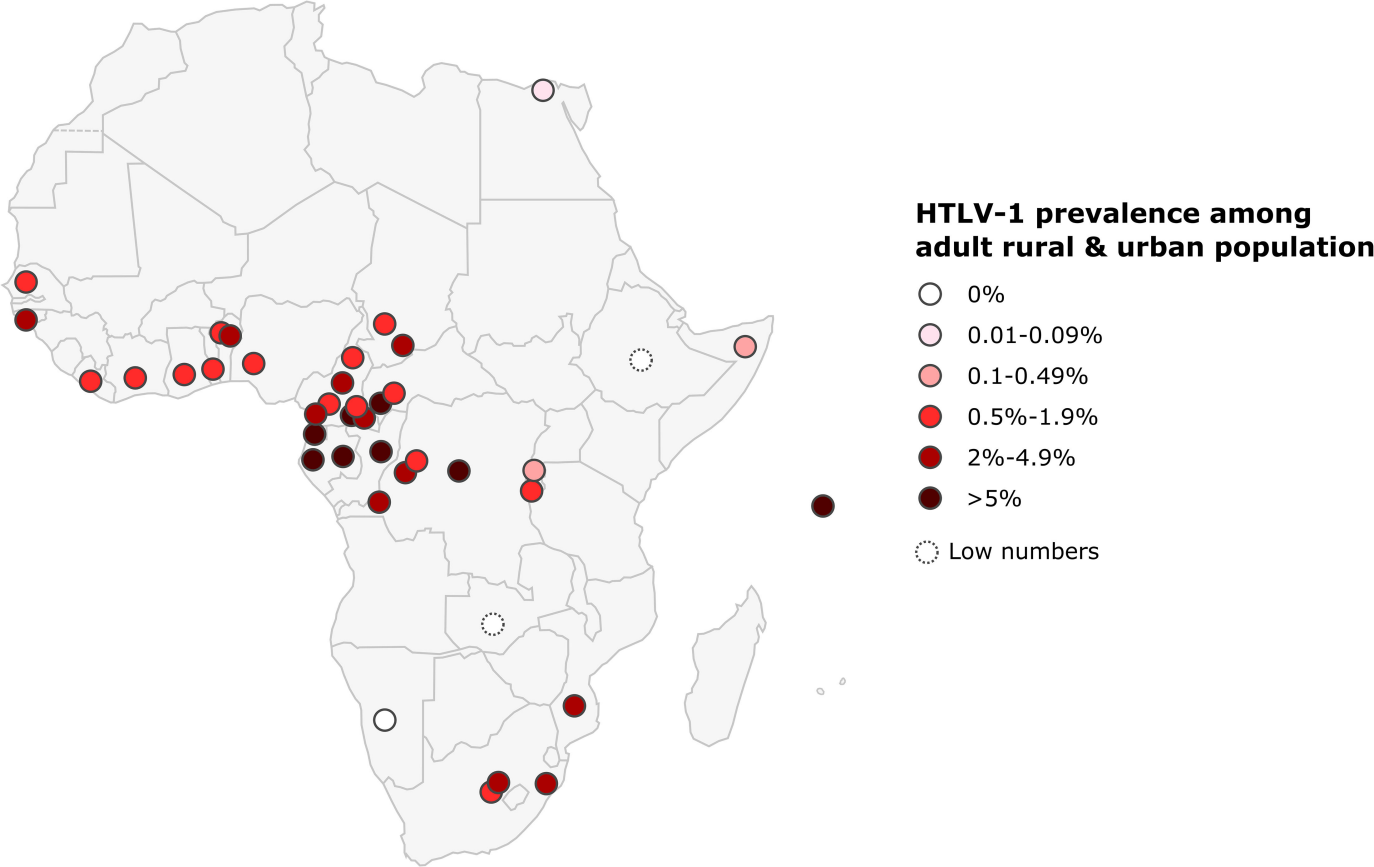
Map of Africa showing location and results of HTLV-1 prevalence studies among rural and urban adult population. The range of HTLV-1 prevalence is indicated by color of red gradient. In case of great geographical or inter-studies variations in prevalence within a country, each result was represented separately at the site of the study location (when available). Studies with low numbers (below 150) are indicated with a dashed line.

Only one study was conducted on a rural population of adult outpatients in North Africa: it reported a prevalence of 0.06% in Egypt. In West Africa, the prevalence of HTLV-1 in the rural adult population generally ranged from 0.7 to 1.7%, but was much higher in Benin and Guinea-Bissau, where it ranged from 1.5 to 4.5% and from 4.6 to 5.2%, respectively. In West Africa, HTLV-1 prevalence was lower in urban populations, ranging from 0.5% in Nigeria to 2.7 to 4.4% in Guinea-Bissau (**Figure 6** and **Table 3**). In Central Africa, HTLV-1 prevalence varied considerably, ranging from 0 to 10.5% in the rural adult population and from 3.1 to 6.6% in the urban population. In East Africa, the prevalence of HTLV-1 was 0% in Zambia, Ethiopia and Namibia, and ranged from 0.1 to 2% in Burundi, Ethiopia, Mozambique, Rwanda, Somalia and Zambia, reaching 6.2% in the Seychelles. In Southern Africa, HTLV-1 prevalence was 1.1% in the rural population and ranged from 0 to 2.6% in cities and was higher in the black population than in white population.

**Table 3:** HTLV-1 prevalence among African rural and urban adult population.

		Rural population			Urban population		
Subregion	Country	HTLV-1 prevalence	N rural	References	HTLV-1 prevalence	N urban	References
Northern Africa	Egypt	0.06%*	3158	(70)			
Western Africa	Benin	1.5-4.5%	6636	(53, 71, 72)			
	Côte d’Ivoire	0.7-1.7%	2686	(31, 54, 73)			
	Ghana	1.3-1.4%*	2567	(35, 74)			
	Guinea	1.10%	2285	(75)			
	Guinea-Bissau	4.6-5.2%	5396	(76, 77)	2.7-4.4%	6590	(78–81)
	Liberia	1.60%	620	(4)			
	Nigeria	0.80%	1640	(82)	0.50%	385	(83)
	Senegal	1.20%	993	(4)			
	Togo	1.20%	1717	(84)			
Central Africa	Cameroon	0.7-6.6%	10975	(17, 18, 40, 85–91)			
	CAR	0-7.4%	2096	(85, 92, 93)			
	Chad	0.5-2%	666	(94)			
	DR of the Congo	1% - 5%	4463	(73, 95, 96)	3.10%	839	(97)
	Equatorial Guinea	10.40%	401	(94)	6.20%	391	(94)
	Gabon	5.3-10.5%	8160	(87, 98–100)	5-6.6%	571	(87, 101)
Eastern Africa	Burundi	1.30%	519	(102)			
	Ethiopia	0%	225	(103)			
	Mozambique	2%	752	(104)			
	Rwanda	0.30%	742	(105)	0.20%	1870	(105)
	Somalia	0.09%	1133*	(106)			
	Zambia	0%	226	(107)			
Southern Africa	Namibia	0%	704	(108)			
	Rep. of South Africa	1.10%	178	(109)	0-2.6%	1268	(109, 110)
Indian Ocean	Rep. of Seychelles	6.20%	1055	(111)			

CAR, Central African Republic; DR, Democratic Republic; Rep, Republic.

*outpatients’ population.

As reported for other endemic areas endemic in the world, HTLV-1 infection is unevenly distributed. Prevalences in rural areas tend to be higher than in urban populations, but geographic variation is often observed in countries with high levels of endemicity for HTLV-1. Thus, in Gabon, HTLV-1 prevalence varies between regions, ranging from 5% to 14% (94, 98, 99). The different prevalences of HTLV-1 are indicated by separate dots placed on a map, corresponding to the site of the survey when possible, to provide a better appreciation of these variations (**Figures 4**–**6**).

### 2.3 Challenges in the evaluation of HTLV-1 prevalence

As exemplified above, it is difficult to estimate the overall prevalence of HTLV-1 prevalence for individual countries or regions of Africa due to the considerable variation of HTLV-1 prevalence between studies. Some of these variations may be due to the differences in the nature of the confirmatory assay used or evolutions in these same assays over the years, limiting the comparability of HTLV-1 prevalence values over time. Another significant variable is the demographics of the study population, as the mean age distribution and sex ratio of the population can have a major impact on prevalence (see Introduction, points 2 and 3). Age- and sex-adjusted prevalence values would provide a more accurate comparison, but it was not possible to extract these data, as age and sex data were missing in several studies. Moreover, some surveys performed on small study populations (fewer than 200 people) were included as no other studies were available for the countries concerned, but the prevalence values obtained in such small studies must be interpreted with care. There is a critical need for studies in areas for which few data are available.

## 3 Risk factors for HTLV-1 infection and modes of transmission

Few epidemiological studies have assessed the risk factors for HTLV-1 infection in Africa. In most studies, the principal limitation is the absence of a multivariable approach. As a result, the proportion of transmission attributable to each mode of transmission and the associated risk factors remain largely unknown in Africa.

### 3.1 Sexual transmission

By contrast to other endemic areas, such as Japan, the USA and the Caribbean region, the sexual transmission of HTLV-1 has rarely been investigated in Africa, even though this mode of transmission is probably the most relevant (**Table 4**). Indirect evidence for this assertion is provided by the increase in HTLV-1 prevalence with age, and higher rates in women, with further support from the association between high-risk sexual behaviour and HTLV-1 infection reported in some studies. In Kinshasa, the prevalence of HTLV-1 was 9.7% in female sex workers (FSWs), but only 2% in pregnant women (42). A higher prevalence of HTLV-1 has been reported among FSWs in DRC (42, 115), Nigeria (82, 116), Guinea-Bissau (117), Gambia (118), Cameroon (119, 120), Djibouti (121) and Côte d’Ivoire (31). High prevalences have also been reported among the clients of FSWs in Djibouti (121) and Guinea-Bissau (117), and in patients with sexual transmitted infections (STIs) in Guinea-Bissau (117), Nigeria (82, 122), Djibouti (121) and South Africa (123). HTLV-1 infection has also been associated with HIV-2 infection in Guinea-Bissau (76, 78, 79, 112).

**Table 4:** Cross-sectional studies of HTLV-1 transmission risk and associated risk factors.

Factors associated with HTLV-1 infection	Countries (references)	
	in multivariable analyses	in univariable analyses
Sexual transmission		
History of prostitution		Guinea-Bissau (78)*
Genital ulcers	Guinea-Bissau (80)*	
Nb of partners	Guinea-Bissau (79, 80)*	
HIV-1 co-infection	Guinea-Bissau (79)*	Guinea-Bissau (77)
HIV-2 co-infection	Guinea-Bissau (78, 79)	Guinea-Bissau (76, 112)
Parenteral transmission		
History of transfusion	Guinea-Bissau (79)**; CAR (92)	Cameroon (86); Gabon (98, 99, 113)
History of intramuscular injection	CAR (92)	
History of intravenous injection	DRC (97)	
History of hospitalization	Gabon (99)	Gabon (98)
History of surgery	Cameroon (86)	Gabon (98)
Ornemental scarification	Guinea-Bissau (79)*	Guinea-Bissau (112)
Zoonotic transmission		
NHP bite	Cameroon (86)	Cameroon (114); Gabon (99); Guinea-Bissau (112)

*Associated factor among women only; ** associated factor among men only.

### 3.2 Maternal transmission

Mother-to-child transmission is also a major mode of HTLV-1 transmission, given the high prevalence in pregnant women in most African countries and the lack of alternatives to breast milk. The rate of mother-to-child transmission has been estimated at 17.5% in Gabon (124), 22% in Gambia (33) and 25% in Guinea-Bissau (125).

### 3.3 Parenteral transmission through blood transfusion

HTLV-1 can be transmitted *via* contaminated blood products, during blood transfusion, organ transplantation or the intravenous injection of drugs. The lack of systematic screening for HTLV-1 infection in African blood banks and the observed prevalence among blood donors indicate that there is a significant risk of contamination *via* blood. An association between blood transfusion history and HTLV-1 infection has previously been reported in Central African Republic (CAR) (92) while in Guinea-Bissau this association was only found in earlier studies and has not been confirmed in more recent ones (79) (**Table 4**). The risk of HTLV-1 infection through blood transfusion was estimated to be as high as the risk of mother-to-child HTLV-1 transmission in children hospitalized in Gabon (113). In South Africa, a case of HAM/TSP was described in a patient who underwent transfusion with contaminated blood (65).

Despite the risk of HTLV-1 transmission and secondary HAM/TSP development, routine screening is not widely implemented in Africa due to its low cost-effectiveness in countries with high endemicity but low-income (126).

### 3.4 Nosocomial transmission through injection, hospitalisation, or surgery

In addition to blood transfusion, a history of hospitalization, surgery or injections has also been shown to be independently associated with HTLV-1 infection in various studies in Central Africa (**Table 4**). For instance, prophylactic intramuscular injections campaigns against trypanosomiasis between 1947 and 1953 are suspected to have simultaneously spread HTLV-1 and hepatitis C virus in CAR (92). These results may reflect nosocomial transmission that occurred decades ago, due to poor disinfection and the reuse of equipment. However, the possibility of current nosocomial transmission remains an open question and cannot be ruled out in some African countries.

### 3.5 Zoonotic transmission

The zoonotic transmission of STLV-1 from non-human primates (NHPs) has only been demonstrated clearly in Central Africa, where bushmeat hunting is frequent and the presence of STLV-1 in monkeys and apes appears to be high. Zoonotic transmission seems to occur primarily through bites from NHPs, the risk being particularly high for gorilla bites (86, 114). NHP bites have been identified as a risk factor in the rural populations of Cameroon and Gabon (86, 99, 114). However, indirect contact with NHPs, such as hunting, injury, cutting or eating bushmeat, has not been identified as a risk factor (73, 86, 95, 99).

### 3.6 HTLV-1 in different ethnic groups

HTLV-1 prevalence can differ between ethnic groups. For example, it is higher in Pygmies than in Bantus in Central African countries (11, 86, 99, 114), and higher in the black population than in the white population in the Republic of South Africa (65, 109). The origin of these ethnic differences remains poorly understood. They probably result from founder effects and have been maintained by higher rates of transmission in these populations, due to a longer duration of breastfeeding, for example (127). Cultural practices, such as ornamental scarification initiation rites and traditional healing practices leading to blood contamination, may represent a risk for HTLV-1 transmission, although associations with HTLV-1 have rarely been reported (112).

## 4 HTLV-1-associated diseases

### 4.1 What do we know about adult T-cell leukemia in Africa?

We know very little about adult T-cell leukemia in Africa. Indeed, only a few cases in African patients have been reported to date (**Tables 5**, **S2**). This low reporting partly reflects the rarity of the disease, but is likely compounded by the very low awareness of HTLV-1 and ATL among local clinicians and by the absence of diagnostic tools (point 4). Such tools are needed to establish an accurate differential diagnosis between ATL and other hematologic malignancies, including cutaneous T-cell lymphomas, such as mycosis fungoides (184, 207, 208) or Sézary’s syndrome (133, 209), which are also present in African patients. An extensive search of medical publications over a period of almost 40 years identified only about 160 reported cases of ATL in patients of African origin (**Table 5**). This figure is ridiculously low for the African continent, which is home to several (two to five) million people infected with HTLV-1. Based on an estimated incidence of ATL of about 1-2/1,000 HTLV-1 carriers (210), a few thousand cases of ATL would be expected to occur annually in African patients. A similar, but less dramatic, situation has already been reported in other highly endemic regions, such as Brazil (211), although to a lesser extent. Indeed, the number ATL cases reported in the national cancer registry in Brazil was 12 per year, compared to the estimated 800 cases per year. Only specific, targeted research on this rare disease will allow a better appreciation of the true incidence of ATL.

**Table 5:** HAM/TSP and ATL cases reported in 33 African countries and Indian Ocean Islands.

African region/Country	ATL case	Reference	HAM/TSP case	Reference	Population (2022 est.)
**North Africa**					
Algeria	1	(128)	-	-	44,178,884
Mauritania	3	(129–131)	2	(128, 132)	4,161,925
Mali	10	(130, 132–135)	1	(132)	20,741,769
Morocco	3	(132, 136, 137)	7	(138, 139)	36,738,229
Unidentified country	2	(140, 141)	-	-	
**North-East Africa**					
Egypt	1	(142)	2	(143)	107,770,524
Sudan	1	(144)	-	-	47,958,856
**West Africa**					
Burkina Faso	1	(132)	-	-	21,935,389
Côte d’Ivoire	21	(130, 132, 135, 136, 145–147)	11	(130, 132, 148–152)	28,713,423
The Gambia	1	(153)	-	-	2,413,403
Ghana	3	(132, 154, 155)	1	(130)	33,107,275
Guinea	3	(132)	1	(156)	13,237,832
Guinea-Bissau	1	(135)	-	-	2,026,778
Liberia	1	(142)	1	(157)	5,358,483
Niger	–	-	1	(158)	24,484,587
Nigeria	12	(3, 132, 159–163)	3	(130, 132)	225,082,083
Angola	1	(130)	–	-	34,795,287
Senegal	28	(129, 130, 132, 164–169)	16	(129, 130, 132, 135, 149, 170–172)	17,923,036
Sierra Leone	1	(173)	–	-	8,692,606
Togo	2	(132)	9	(84)	8,492,333
Unidentified country	1	(174)	–	-	
**Central Africa**					
Central African Republic	–	-	3	(171, 175, 176)	5,454,533
Chad	–	-	2	(130, 135)	17,963,211
Dem. Rep. of the Congo	1	(177)	56	(130, 135, 171, 175, 178–183)	108,407,721
Gabon	6	(136, 184, 185)	8	(87, 135, 186)	2,340,613
Republic of the Congo	–	-	1	(130)	5,546,307
**East Africa**					
Ethiopia	–	-	4	(187–189)	113,656,596
**Southern Africa**					
Eswatini (ex-Swaziland)	–	-	1	(130)	1,121,761
Mozambique	–	-	4	(190)	31,693,239
Republic of South Africa	36	(191–195)	202	(65, 109, 110, 135, 190, 196–200)	57,516,665
Zambia	–	-	1	(130)	19,642,123
Zimbabwe	–	-	4	(63, 130)	15,121,004
**Africa**					
Unidentified country	15	(201, 202)			
**Indian Ocean Islands**					
Comoros	–	-	1	(130)	876,437
Republic of Seychelles	1	(203)	15	(204, 205)	97,017
The Reunion Island	2	(206)	4	(67, 69, 206)	868,800

The few cases of ATL identified locally were mostly performed in South Africa, Senegal, Nigeria, Gabon and, to a lesser extent, in Mali (**Table 5**). Teams from the Principal Hospital in Dakar, the dermatology department of Thiès Hospital in Senegal, and the Institut Pasteur in Dakar have reported about 20 cases of ATL over a period extending from 1994 to 2010 (129, 164–168, 212). In Nigeria, several small series of patients with ATL hospitalized in Ibadan and Zaria have been reported, but these studies are a quite dated (3, 159–161). In Gabon, a sparsely populated country with high endemicity for HTLV-1, studies have only reported a few cases of ATL hospitalized in Libreville in the 1990s (136, 184, 185). In Mali, rare cases of ATL have been diagnosed at the Institut Marchoux in Bamako (133, 134), together with other cutaneous T-cell lymphomas, such as Sézary syndrome (133). Surprisingly, only 36 cases of ATL have been diagnosed in South Africa despite the high level of endemicity of HTLV-1, and the presence of a hospital system and virology research facilities that are much developed than those in many other sub-Saharan African countries (191–195).

Little is known about the situation in East Africa, but because some patients suffering from other types of cutaneous T-cell lymphomas have been reported in Rwanda (209) and Tanzania (207), we can assume that ATL is extremely rare in this region of low HTLV-1 endemicity (144).

The vast majority of cases of ATL in patients from Africa reported in the medical literature were diagnosed in France, England and the United States, in immigrants and patients repatriated for medical reasons (**Tables 5**, **S2**). In France, a large proportion of the African patients with ATL reported mostly in recent years were diagnosed in Paris and originated from the Côte d’Ivoire (130, 132, 135, 136, 145). In addition, a small series of patients from Mali and Senegal have been diagnosed and hospitalized in France since the 1990s. More recently, a few cases of ATL in patients from Nigeria were diagnosed in the United States, England, Canada, and Italy (161, 162). Finally, sporadic cases of ATL have been reported in patients from other West African countries, such as Sierra Leone, Guinea, Guinea-Bissau, Gambia, Burkina Faso, Ghana, and Togo (132, 135, 153, 173). For North Africa, where little is known about the prevalence of HTLV-1, only a few cases of ATL have been reported in patients from Morocco, Mauritania, and Algeria hospitalized in France (128–130, 132, 136, 137).

Interestingly, the mean age of ATL cases in Africans is about 40-50 years, similar to that of patients in Brazil and the Caribbean, but very different from that in Japan, where the mean age of ATL cases is 60-70 years old (213). This disparity between regions suggests the existence of cofactors, such as other infections or differences in population genetics, which could favor the development of ATL at a younger age.

**Figure 7:**
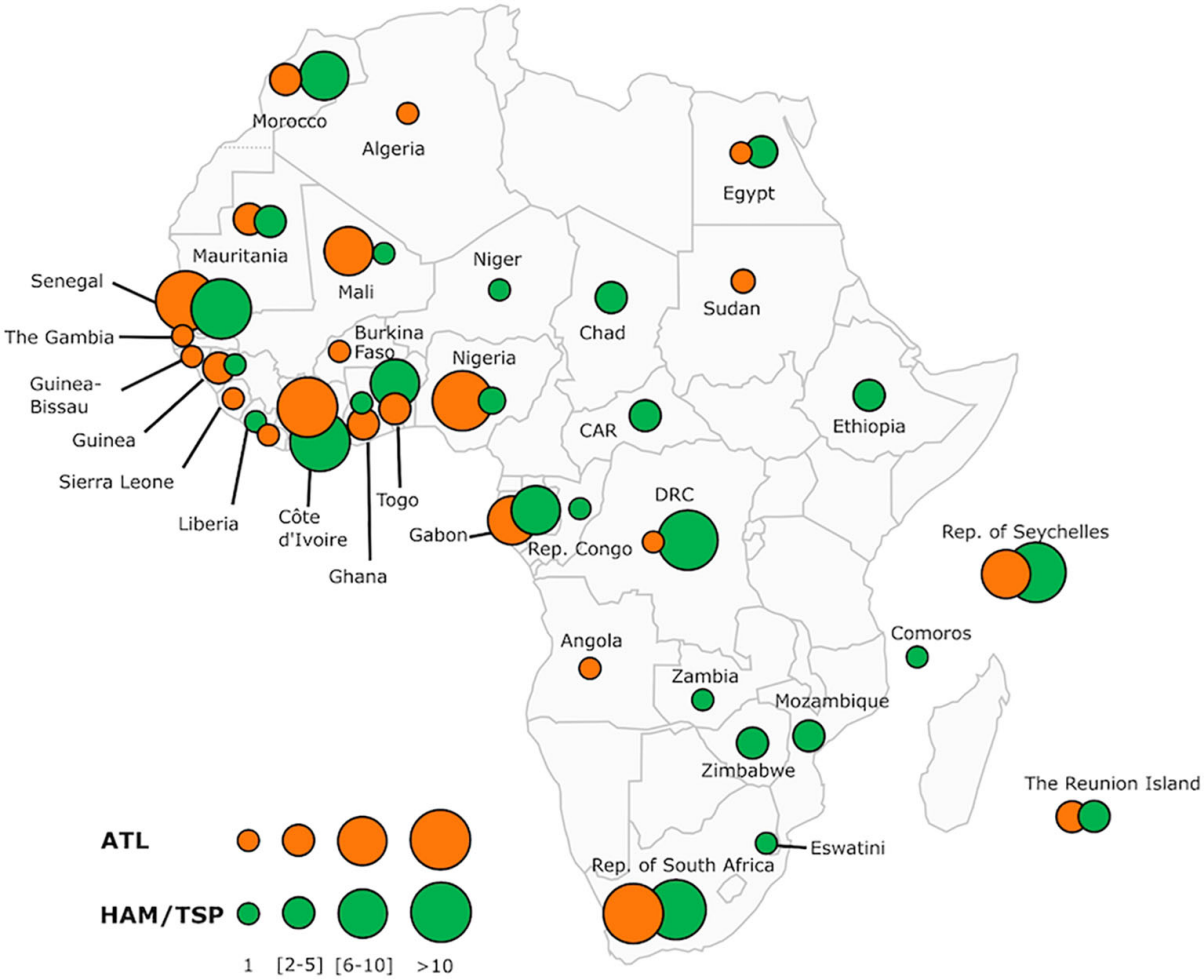
Map of Africa showing the overall distribution of two major HTLV-1-associated diseases: adult T-cell leukemia/lymphoma (ATL) and tropical spastic paraparesis or HTLV-1 associated Myelopathy (HAM/TSP) reported across the continent and some Indian Ocean islands (Comoros, Seychelles and The Reunion Island). The number of ATL and HAM/TSP cases is shown for each African country and archipelagoes. The map includes information from articles available on PubMed. We also included most of the book chapters and abstracts published at the 20 International Conferences on HTLV and related viruses held since 1985. Countries without indication do not have informative published data on these two HTLV-1 associated diseases. The size of the circles is proportional to the number of reported cases. The smallest size corresponds to 1 reported case, intermediate sizes to a maximum of 5 or 10 cases and the largest to a minimum of 11 cases. Of the 34 countries and islands for which we have records, only 16 have reported cases available for both diseases. DRC, Democratic Republic of The Congo; CAR, Central African Republic; Rep, Republic.

The published cases of ATL in patients of African origin are listed in the **Supplemental Table 2** and illustrated in **Figure 7**. A few other cases or series of ATL in patients of African descent have been reported, but with an important lack of data, making it impossible to include them in this table.

### 4.2 What is the situation for HTLV-1-associated myelopathy/tropical spastic paraparesis in Africa?

Data for HAM/TSP in African patients are also very fragmentary, with both a significant lack of awareness of this disease among local clinicians and a lack of means for diagnosing HTLV-1 infection. Moreover, when considering neurological diseases, the scarcity of neurologists and of biological and technical means, such as radiological explorations (scans and MRI) in several African countries represents a major handicap. Indeed, it is crucial to differentiate the diagnosis of HAM/TSP from other spastic paraplegia-like syndromes, which are quite common in Africa. Finally, many patients with progressive gait disturbance do not, or cannot seek medical attention. The rare studies on HAM/TSP were performed in capital cities, and there are almost no data available for rural populations. This may account for the considerable underreporting of HAM/TSP in Africa, despite its chronic nature.

Only a few relatively large series of HAM/TSP cases in African patients have been published by or in association with local African physicians (**Tables 5**, **S3**). These cases were reported in the early 1990s, in the Republic of South Africa (RSA) and in Zaire (now the DRC).

The first of these studies was performed in RSA, on a series of 24 patients, mostly women, aged between 40 and 50 years, from the Durban area of Natal Province (196). A subsequent study led to the reporting of a series of 90 cases of HAM/TSP diagnosed within a few years of follow-up (110). Apart from a few published sporadic cases, including cases of HAM/TSP developing after blood transfusions, there have, surprisingly, been no new data reported on HAM/TSP since, in this region of South Africa where HTLV-1 is highly endemic.

The second study described a series of 25 HAM/TSP patients hospitalized in Lisala, Equateur Province in DRC. Most of the patients were women, aged 30-40 years at onset (178). Another study from DRC identified six cases of HAM/TSP in Inongo (179). Interestingly, these studies reported ethnic and familial clustering, suggesting the presence of various cofactors (genetic or environmental, including cultural habits).

The other four countries in continental Africa in which studies on smaller series of HAM/TSP cases have been performed locally are Côte d’Ivoire, Senegal and Togo in West Africa, and Gabon in Central Africa (**Tables 5**, **S3**). Tropical neuromyelopathies of unknown origin are frequent in Côte d’Ivoire, and several studies conducted in the 1990s demonstrated the presence of HTLV-1 infection in small series of patients with chronic pyramidal syndrome diagnosed as HAM/TSP (148). In a series of 13 patients infected with HTLV-1, several were coinfected with HIV-1. In Senegal, a series of six cases of typical HAM/TSP was reported in 1995 in the neurology department of the hospital in Dakar (129). Similarly, a study performed in the late 1980s in the neurology department of the hospital in Lomé, Togo, reported a series of nine cases of HAM/TSP (84). Four cases were also reported in Gabon in the 1990s (87, 186). A few other sporadic cases of HAM/TSP have been reported in patients from Morocco and Ethiopia (138, 139, 187, 188).

In Southern Africa, besides the cases reported in RSA, a few cases have been reported in Mozambique, Zimbabwe, Zambia and Eswatini (formerly Swaziland).

Most of the other reported cases of HAM/TSP in African patients were diagnosed in France or England, in immigrants or repatriated patients (**Table S3**). These few sporadic cases of HAM/TSP have been reported in patients from West African countries, such as Senegal, Mali, Côte d’Ivoire, Liberia, Ghana and Nigeria, and from Central African countries, such as DRC, Gabon, Congo, CAR, and Chad. One case has been reported in a patient from Mauritania (128).

We also included studies from the Seychelles, an archipelago in the Indian Ocean with a history and settlement closely linked to the eastern coast of the African continent, in this review. In 1987, a cluster of 17 cases of HAM/TSP was reported in patients living in the Seychelles (204). Finally, a few cases of HAM/TSP have been reported in patients from The Reunion Island and the Comoros, also located in the Indian Ocean and with strong connections to mainland Africa (67, 206).

### 4.3 Infective dermatitis in Africa

Infective dermatitis (ID) is a rare dermatological condition associated with HTLV-1 infection that was initially described in Jamaican children in 1990 (214). It has since been reported in other endemic countries, mostly in Jamaica and Brazil, with a few sporadic cases diagnosed in French Guiana, Guadeloupe, Colombia, Trinidad, Japan, and Central Australia.

We have reported the first cases of ID in African patients in 2004 (212). Over a three-year period, five of the approximately 2,500 children seen at a dermatological center in Dakar, Senegal, were diagnosed with typical ID associated with HTLV-1 infection. The oldest patient was 17 years old and had also developed chronic ATL. Nearly 10 years later, a series of 19 cases of ID were reported in KwaZulu Natal, South Africa (215). This series of patients is from a three-year study in an outpatient facility managing more than 3,000 patients with various skin conditions. These results, once again, support the hypothesis of a significant underreporting of this disease.

Several cases of HTLV-1-associated uveitis and myositis have been reported in Sierra-Leone, Togo, and Côte d’Ivoire (216, 217).

Unfortunately, very little progress has been made over the last two decades towards understanding the clinical and epidemiological aspects of HTLV-1-associated diseases in Africa. The prevalence, incidence, and geographic distribution of these diseases, which are globally neglected in Africa, remain almost unknown to this day. Only major efforts to inform and sensitize local medical staff — both clinicians and the personnel of biology and virology laboratories — can improve our knowledge of this virus and associated diseases on this continent.

## 5 Molecular epidemiology of HTLV-1 in Africa

### 5.1 Genetic diversity

**Figure 8:**
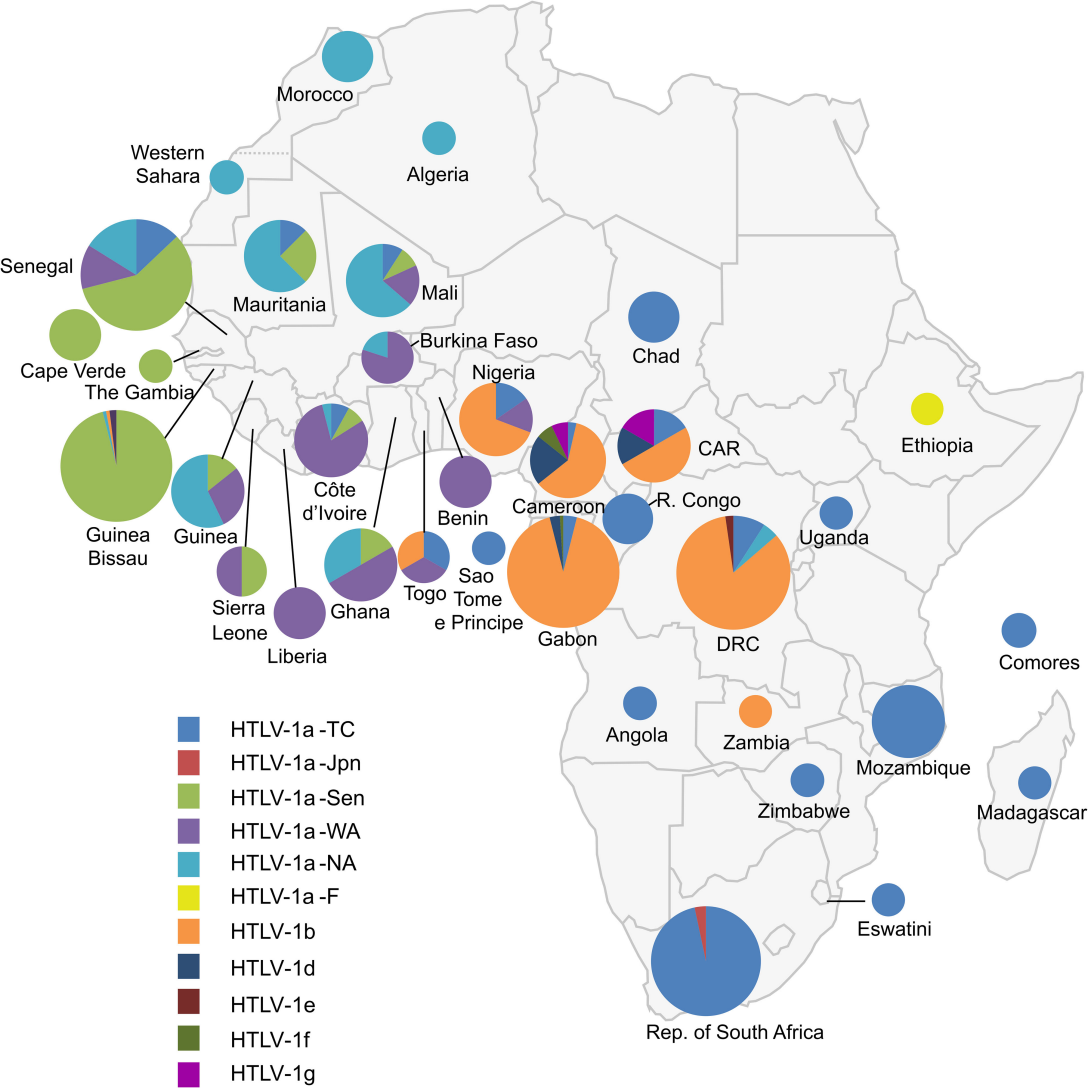
Map of Africa showing the general distribution of HTLV-1 genotypes across the continent [Source: Afonso et al., Retrovirology, 2019 – (218)]. The proportion of the different HTLV-1 genotypes and subgroups is presented for each African country. This figure incorporates the information from 24 papers of molecular epidemiology available on PubMed. It also incorporates results from one manuscript in preparation (Filippone C. et al.) concerning the situation in Madagascar. Countries without indications have no informative published data on HTLV-1 genotypes between 1994–2019. The size of the circles is proportional to the number of strains identified. The smallest size corresponds to 1 characterized strain, the intermediate sizes to a maximum of 5 or 30 strains and the largest to a minimum of 30 strains. HTLV-1a-North African (HTLV-1a-NA), HTLV-1a-Senegalese (HTLV-1a-Sen), HTLV-1a-West African (HTLV-1a-WA), HTLV-1b and HTLV-1a-Transcontinental (HTLV-1-a-TC) are the most common throughout the continent in North, West, Central and the Austral parts respectively. HTLV-1d, -e and-g have been identified in Central Africa (Cameroon and Gabon) and -f in East Africa (Ethiopia).

**Figure 9:**
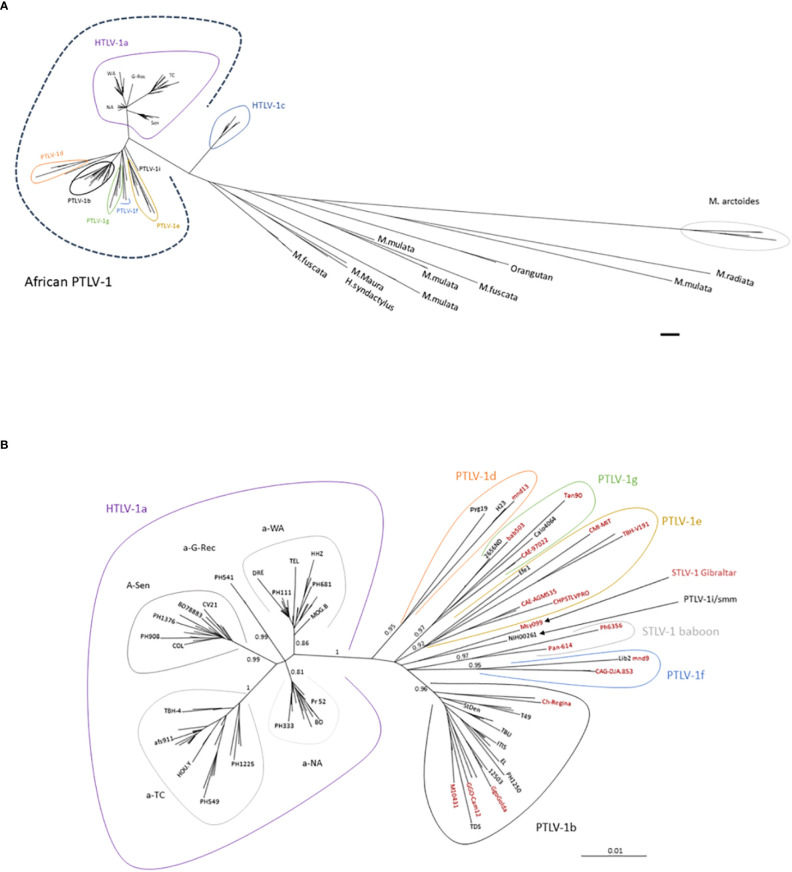
**(A)** General disposition of African PTLV-1 among the other PTLV-1. Phylogenetic tree generated from a 711-nucleotide long alignment of 196 sequences. Sequences comprise African PTLV-1, Australo-Melanesian HTLV-1c and Asian Macaque STLV-1 strains. The phylogenetic tree was generated using the Maximum Likelihood method. The scale bar represents nucleotide substitutions per site. **(B)** Phylogenetic tree presenting the genetic diversity of African PTLV-1. The analysis was performed on an alignment of 125 HTLV-1 and 30 STLV-1 sequences (presented in black and red respectively). This alignment contains distinct African HTLV-1 full-length LTR sequences (in black), and the most representative STLV-1 full-length sequences (in red). The size of the complete LTR available in GenBank varies between 745-nt and 790-nt depending on the strain, resulting in an alignment of 772-nt nucleotides (due to difficulties in aligning some regions). The phylogenetic tree was derived by the neighbor-joining method using the GTR model (gamma = 0.5017), with the PAUP software. Horizontal branch lengths are drawn to scale, which is indicated by the bar. The topology of the phylogenetic tree was validated by Maximum Likelihood (using the SeaView5 software), and the strength of the branches was estimated using approximate likelihood-ratio test (aLRT). The values correspond to the calculated probability. The definition of genotypes (or subtypes of PTLV-1) depends on the number of sequences present for each group, and the size of the sequences. The historically coined “South African and East African STLV-1” subgroup (219) is now presented as part of the PTLV-1e genotype. Similarly, the “Central and West Africa STLV-1” subgroups now belong to the HTLV-1g genotype. All genotypes represented in Africa are presented in the tree, except HTLV-1h (as described by Liégeois (220), for which only partial LTR sequences are available. HTLV-1a is strictly present in humans, STLV-1 Gibraltar and STLV-1 baboon have never been detected in humans. All the other subgroups have been identified both in human and simian hosts.

The genetic diversity of HTLV-1 is the greatest in Africa, where six distinct genotypes (a, b, d, e, f, g) have been identified (**Figures 8**, **9A, B**) (218). These genotypes are generally related to the geographic origin of the infected individuals. Furthermore, clades can be detected within a given genotype. These intragenotype geographically-related clades probably arose from a founder effect: a bottleneck in evolution followed by speciation of genetically isolated populations.

The most frequent genotype in Central Africa is genotype b (**Figure 8**). It accounts for 70-100% of the strains from Gabon, Cameroon, DRC, and Nigeria. For example, in Gabon, more than 200 of the almost 230 reported viral strains belong to genotype b (98). In DRC, a recent study found that all 107 HTLV-1 strains belonged to genotype b (95). Interestingly, there is some diversity within genotype b, with different phylogenetic clades that seem to be linked to geographic origin. The other genotypes found in Central Africa (d, e, f and g) are usually rare, although genotype d viruses accounted for 3% to 5% of genotypes in two large studies in Gabon (98, 221). By contrast, only a few, sporadic of strains of genotypes e, f and g have been described in Gabon (222) and Cameroon (15, 114, 223). Of note, viruses of genotypes b and d have been reported both in individuals of Bantu origin and in Pygmies (114, 135, 221, 223).

The vast majority of HTLV-1 strains from West and North Africa belong to genotype a (which is known to be ‘Cosmopolitan’, with only a few exceptions from Côte d’Ivoire, where a small number of genotype g strains were reported, together with specific local strains (i/sm) (73, 157, 224, 225). The a-genotype strains isolated in North and West Africa form five clades (Figs. 8-9). The distribution of these clades roughly reflects the geographic origin of the infected individuals (132). The North African subgroup (a-NA) was mostly found in individuals originating from Morocco, Mauritania, Western Sahara, Algeria, and Mali. The West African (a-WA) subgroup was mostly found in populations from Côte d’Ivoire, Liberia, Ghana, Burkina Faso, and Benin. Strains from the HTLV-1 a-WA clade are also highly endemic to Noir-Marron populations living in Suriname and French Guiana, probably as a result of the historical transatlantic slave trade. This finding clearly confirms that the low genetic variability of HTLV-1 and validated the use of HTLV-1 as a marker of migration in infected populations (226). Strains from Senegal and neighboring countries (Guinea-Bissau, Gambia, Mali and Cabo Verde) mostly belong to the a-Sen clade. The lower abundance of this subtype outside its probable initial focus, probably testifies to the migrations and population movements still frequently occurring today between the inhabitants of Senegal and other West African countries, such as Côte d’Ivoire, and Ghana. We recently showed that the a-Sen clade arose from recombination between a-WA and a-NA strains (130). Furthermore, some strains present in Guinea and Ghana coalesce into a subgroup named a-G-rec, which also arose through recombination between a-WA and a-NA strains (see the next section for more details) (132).

When considering Southern Africa, many strains have been sequenced, but they are almost exclusively originate from South Africa (227) and Mozambique (228). There are only rare strains available from neighboring countries (Zimbabwe, Eswatini (formerly Swaziland), Madagascar, etc.) (63, 130). Almost all of these sequences belong to a subgroup of a-genotype called ‘Transcontinental’ (a-TC). This subgroup corresponds to the most common and most widely disseminated clade of HTLV-1 in the world. Indeed, it is also present in many highly endemic regions of South America, such as Brazil and Peru, where it constitutes the major subgroup, but also in North America (the USA and Canada), and Japan. The wide spread of the Transcontinental subgroup throughout the world seems to be linked, above all, to past movements of infected populations, particularly through the slave trade. Thus, certain strains from Mozambique and South Africa are very closely related to each other, and to those present in Brazil, testifying to a probable common origin in Southern Africa (229). In Mozambique, two other clades within HTLV-1 a-TC are present. The sequences of one clade segregate with strains found in the Middle-East and in India, and are probably related to the past migration of Indian populations to Maputo. The other clade seems to be more specific to Mozambique and probably reflects local speciation (228).

### 5.2 Origins of this HTLV-1 diversity

It is currently thought that HTLV-1 viruses have derived from simian counterpart, STLV-1. These viruses spread *via* several routes in monkey colonies *in natura*: sexual transmission, mother-to-offspring transmission, through wounds acquired during fights for sexual dominance, and during the consumption of infected prey (230–234). STLV-1 can cause clonal proliferations of T-cells *in vivo;* cases of ATL have been described in many African and Asian simian species (233, 235).

In Africa and Asia, more than 25 species of non-human primates, both monkeys (*Cercopithecus*, baboons, mandrills, macaques, etc.) and apes (chimpanzees, gorillas, orangutans, gibbons), are naturally infected with STLV-1 (236). Many African HTLV-1 strains have very similar simian homologs, suggesting recent zoonotic transmission. Moreover, the zoonotic transmission of STLV-1-b was strongly suggested by case-control study studies in Gabon and Cameroon conducted on hunters bitten by gorillas (114, 221). In Cameroon, the prevalence of HTLV-1 increased significantly with bite severity, strongly suggesting a causal relationship between the bite and interspecies viral transmission involving contact between the saliva of the monkey or ape and the blood of the hunter (114). Bite-related interspecies transmission has been demonstrated even more clearly in the context of other retroviruses present in the saliva of African apes, the simian foamy viruses (237, 238). Like HTLV-1b, HTLV-1d seems to be transmitted both between humans, and from NHPs to humans, probably through bites from infected animals (221). STLV-1d is endemic in mandrills and *C. nictitans* (220, 236, 239). HTLV-1 genotypes e, f, and g are sporadically detected in association with hunting accidents involving small monkeys naturally infected with these genotypes. Finally, other studies in Côte d’Ivoire and DRC have also strongly suggested that interspecies STLV-1 (of genotype i/sm or b) transmission occurs from different monkey species (including sooty mangabey and *Allenopithecus nigroviridis*) to humans, particularly after contact with NHPs (219, 224).

By contrast to the situation for the HTLV-1 a-TC and, at a lesser extent, HTLV-1 b genotypes, there is little evidence to suggest that interhuman transmission of the rare e, f, and g genotypes occurs. It would, therefore, be interesting to study further the possible intrafamilial transmission of these viruses. If it is confirmed that these rare genotypes are almost exclusively transmitted to humans from NHPs, it would be interesting to quantify the proviral load in infected individuals, and to determine whether these viruses are rendered less viable as a result of the innate immune responses (such as APOBEC editing). Indeed, a low proviral load (125, 240) or a massive editing of the virus (241–243) could render it less viable and less able to spread between humans.

### 5.3 Unresolved questions

1. What is the origin of the cosmopolitan HTLV-1a genotype? No closely related STLV-1 strain has ever been described. This raises several possibilities. STLV-1a could still exist, possibly with a low prevalence, in one or more simian species that have not yet been studied. The natural host of STLV-1a may have disappeared or the virus may have been cleared from the original host. When considering a molecular clock model, the choice of either hypothesis would generate very different HTLV-1a spillover and diversification dates (see part V).
2. Three African genotypes currently seem to be exclusively simian: STLV-1 baboon (219), STLV-1 Gibraltar (244), which is found in African macaques, and STLV Uganda (245) (for which only *env* sequences are available; these viruses are not, therefore, represented in **Figure 9B**. Despite numerous contacts with infected animals, there are no reports of such strains infecting humans. There are many possible explanations for this. The first is that the prevalence of these viruses is low in the wild, rendering the probability of transmission very low. Alternatively, these viruses may be intrinsically unable to establish chronic infection in humans. The persistence of HTLV-1a *in vivo* depends on the expression of auxiliary proteins: P12/8, P13 and P30 (246–248). These proteins are essential for the propagation and immune escape of the HTLV-1a virus and for the proliferation of infected cells. A recent *in silico* analysis of the available complete genomes showed that the STLV-1e-g and i/sm genotypes do not encode the canonical accessory proteins (249). The identification of human counterparts suggests that there are alternative proteins that render the virus persistent in humans. The Asian macaque STLV-1 viruses lack the three canonical accessory proteins, which may account for their lack of transmission to humans. A similar phenomenon may be at work in the African genotypes not yet detected in humans. The testing of this hypothesis will require the generation of more complete genomes for all HTLV-1 genotypes.

## 6 Evolution of HTLV-1, mutation rate, and limitations to the use of molecular clock models

### 6.1 Difficulties calculating mutation rates

When determining mutation rates for HTLV-1, it is important to bear in mind that there are two major phases of infection (250, 251). During primary infection, the virus spreads by cell-to-cell contact, and reverse transcription occurs. Reverse transcription has an error rate of 7.E^-6^ mutations/base pair/replication cycle (252). This results in the generation of some diversity during primary infection, with proviruses forming quasi-species within the host. A few months later, viremia is no longer detectable, and the levels of markers of recent infection (e.g. 2 LTR DNA circles) decrease. During this second phase, the virus persists during cell division (253, 254). During clonal expansion, the viral genome is replicated by the cellular DNA polymerase, which has a lower mutation rate (about E-8 mutations/base pair/mitosis). However, viral proteins induce genetic instability by inactivating checkpoints and altering the DNA damage repair system [such as the nucleotide excision repair mechanism – (255)]. The mutation rate per cell cycle is, therefore, probably higher. Moreover, HTLV-1-infected cells proliferate more than uninfected cells, mostly due to the promitotic activity of the viral proteins Tax and HBZ (256). The mutation rate for a given period of time (calculated as mutations/bp/year) would, therefore be higher for HTLV^+^ cells than for uninfected cells.

Despite the processes described above, longitudinal studies have reported that the viral genome sequence remains constant over the years (226). This may be due to the sequencing method (which generates a consensus sequence of sequences circulating in the donor, as the PCR product is often not cloned but sequenced in bulk), or the fact that studies are often conducted on short genomic fragments.

Such a genetic stability is even more astonishing as the pressure exerted by the immune response should favor the emergence of escape mutants. HTLV-1 evasion to the immune response is purportedly achieved through repression of viral expression, frequently referred as pseudo-latency, through epigenetic regulation (257–259).

HTLV-1 genome diversification is also limited during human-to-human transmission. The mutation rate has been estimated in several vertical/intrafamilial transmission chain studies. A study in the DRC (formerly Zaire) revealed that 10 related individuals carried an identical virus, with no mutation (in a 755-nt segment of the LTR) (260). In this case, the mutation rate is essentially zero. Another study in South America found two mutations in the LTR (756-bp long) and three mutations in *env* (522-bp long) in 16 vertical transmission chains (260). From this study, one can estimate the mutation rate per transmission chain of about 2E-4 substitutions/site/transmission chain. We can assume that the actual mutation rate per transmission chain probably lies between these two values.

Therefore, the apparent HTLV-1 mutation rate (substitution/site/year) increases with the number of transmission chains (human-to-human transmission) per period. Therefore, in conditions where the virus is frequently transmitted between different individuals, e.g. intravenous drug usage, or nosocomial transmission, or through ritual practices (such as scarification), the mutation rate per year will be higher than in conditions where the number of transmission rates is lower (as vertical transmission through breast-feeding). Of note, due to the limited genetic variability within an individual, as stated above, prolonged breast feeding or recurrent intercourse between two individuals should be considered as a single transmission chain.

Differences in apparent mutation rates depending on the modes of transmission have been previously demonstrated for HTLV-2: HTLV-2 mutation rate was found higher among intravenous drug users then when considering populations where the virus was transmitted vertically or by sexual transmission (261).

Phylogenetic studies have estimated the apparent mutation rate at 2.1E−7 and 8E−7 subst/site/year (262, 263). These values have been obtained using molecular clock models and anchoring dates (often the divergence of HTLV-1c). However, the molecular clock model is based on a major assumption: viruses are considered to evolve mostly *via* continuous accumulation of mutations. The model rarely accounts for saltatory evolution processes, such as recombination.

### 6.2 Recombination occurs in HTLV-1

Until recently, it was believed that HTLV-1 evolution occurs only through genetic drift and mutation. Recombination was ignored in HTLV-1 evolution. This was further comforted by a study on 10 patients, which reported that HTLV-1 infected T-cells contain a single integrated provirus (264).

However, in some cases, proviral load is higher than 100% in ATL patients (265–267), evidencing the possibility of multiple integrations. Moreover, a study presented in an international meeting reported frequent superinfection in HTLV-1 clone T cells (268). Nevertheless, the clones with multiple integration may be rare or short lived, which would make them difficult to detect.

Indirect evidence of multiple infection with different HTLV-1 strains was brought by phylogenetic studies. Indeed, we have identified two viral clades that have emerged through recombination (130). Some strains collected from individuals in North Africa (a-NA) are the result of recombination between HTLV-1 strains related to strains currently present in Senegal (a-Sen) and West Africa (a-WA). In this case, only one point of recombination was identified, in the LTR, at the U3/RU5 junction. The location of this recombination site suggests that recombination occurred during reverse transcription, rather than as a consequence of recombination between 2 proviral copies. Indeed, the junction corresponds to the first shift in RNA template of the reverse transcriptase. In Senegal and Ghana, we also identified another clade (a-G-rec) resulting from recombination between a-WA and a-Sen strains (132). In this clade, we identified two points of recombination, one at the U3/RU5 junction and the other in the *env* gene. It remains unclear why the only recombinant groups identified to date arose through recombination between a-WA and a-Sen strains. It is possible that recombination occurred between these two groups because the infected populations are more mobile and have ended up encountering each other more frequently. Alternatively, not all recombination events may generate functional viruses. The fitness of the generated clade may be low, leading to the disappearance of the other recombinants over time. It seems most likely that the detection of recombination requires the parental groups to be sufficiently divergent. If they are too alike, it may prove difficult to differentiate between strains obtained by mutation and those obtained by recombination. In Africa, there are no clear clades with significant bootstrap values among a-TC strains. It appears likely that recombination has occurred but that there were so few differences between the parental strains that we cannot trace the recombination back to the parental strains. For the identification of such recombinants, we would need more informative positions. The generation of complete genome sequences would, therefore, be the key to identifying recombinant groups.

### 6.3 Dating the emergence of genotypes or subgroups is a tricky process

Several previous studies used the molecular clock hypothesis to date speciation between the different HTLV-1 genotypes (130, 260, 262, 263, 269–271). This dating may yield highly variable results, according to the hypothesis underlying the model and the sequences considered.

First, as mentioned above, evolution rates depend on the mode of transmission (vertical, or through intravenous drug use, for example). Molecular clock methods should be applied only to strains with identical modes of transmission (261). Moreover, if strains are sequenced from ATL samples, they may have acquired mutations due to the genetic instability associated with oncogenesis (272).

Second, molecular clock models are unable to take saltatory evolution phenomena, such as recombination and deletion/insertion into account correctly. We have now demonstrated that recombination occurs in HTLV-1, and may be frequent, rendering the molecular clock impossible to implement within the HTLV-1a genotype.

Third, it is unclear whether STLV has the same evolution dynamics in simian and human hosts. Therefore, when studying genotypes present in both NHPs and humans, the estimated rate may correspond to a weighted average between the rates in the different species.

In light of all these issues, the estimated date of divergence between HTLV-1a and HTLV-1c ranges from 10 to 120 thousand years ago (263, 269, 271). We consider these dates to be highly questionable, given the current state of knowledge of viral dynamics and evolution. We believe that more complete sequences are required to overcome these limitations, to eliminate those generated by recombination, and to have data for more informative positions.

The African continent is currently considered as a highly (if not the highest) endemic area in the world for HTLV-1, with a few million people infected. The distribution of HTLV-1 which remains poorly understood on this continent, appears to be very heterogenous, with high prevalence of HTLV-1 in the central African regions and low prevalence in the eastern and northern regions. There is a huge underreporting of HTLV-1 associated diseases in Africa, including ATL and HAM/TSP.

The number of African people infected with HTLV-1 and developing associated diseases is likely to increase over the next century due to rapidly growing population and the aging of populations (prevalence increases with age), as well as the lack of preventive measures to reduce the spread of HTLV-1.

In order to improve the situation (current and future) and reduce the burden of HTLV-1 in Africa, the priorities are clear and will require both inter-African and North/South collaborations and specific financial support to:

1. Raise awareness through major information campaigns, as has been developed in other endemic areas, such as Brazil and Japan. These campaigns should primarily target healthcare professionals (clinical and laboratory staff) to improve knowledge about HTLV-1 and related diseases, which are still largely neglected by the medical community in many parts of Africa.
2. Promote large-scale studies in general (representative) populations and in hospitals. Such comprehensive studies are essential to assess the prevalence of HTLV-1 infection and the real situation regarding associated diseases.
3. Develop public health measures in areas of high endemicity, with particular emphasis on blood banks to prevent the spread of HTLV-1 by transfusion, which may be associated with the development of HTLV-1 neurological diseases.

## Author contributions

All authors listed have made a substantial, direct, and intellectual contribution to the work and approved it for publication.
