# Supplementary material for: Geographic distribution, clinical epidemiology and genetic diversity of the human oncogenic retrovirus HTLV-1 in Africa, the world’s largest endemic area
Source: Front Immunol. 2023 Feb 3;14:1043600. doi: 10.3389/fimmu.2023.1043600 (PMC9935834; doi:10.3389/fimmu.2023.1043600)
Supplement: Supplementary file 1 [file Table_1.pdf]

Table S1: Reports of HTLV-1 epidemiological studies in Africa from 1984 to 2022

Region	Country	Reference	Exclusion criteria	Study population detailled	Screening assay	Confirmatory assay	Study-period	HTLV-1 prevalence % (n/N)	Mean age or Med. (range)	%male (or sex-ratio)
Northern Africa	**Algeria**	**Larouze et al., 1985**	Other population (SW)	FSW	RIPA	RIPA		0% (0/140)	(18-42)	0%
Northern Africa	**Egypt**	**Saxinger et al., 1984**	Other population (Patients)	Patients	ELISA		NA	2% (2/101)	NA	NA
Northern Africa	**Egypt**	**El-ghazzawi et al., 1987**		BD + else	ELISA + RIPA	RIPA	NA	0.1% (1/866 BD); 0%(0/71 prisoners, 38 DA, 54 patients)	NA	NA
Northern Africa	**Egypt**	**El-Farrash et al., 1988**		Pop : Out-patients	PA	WB (Furijebio)	NA	0.1% (2/3158)	NA	NA
Northern Africa	**Egypt**	**Khalifa et al., 1990**	Other population (Children)	Children	ELISA	None	NA	0% (0/205)		M to F ratio 1.3/1
Northern Africa	**Egypt**	**Constantine et al., 1991**	Low numbers	BD; high risk population (SW, STIs; DA)	ELISA	WB	1987-1988	2.2%(3/133 male BD); (2/279 drug addicts); (1/30 patients with STDs);0% (0 /47 blood recipients); 0% (0/158 SW)	30.8 (16-75)	BD: 98%
Northern Africa	**Morocco**	**De thé et al., 1985**	No confirmation	NA	ELISA x2	None		0.6% (4/677)	(20-49)	NA
Northern Africa	**Sudan**	**Saliheen et al., 2016**	No confirmation	BD	ELISA	None	2013	0.2% (1/540)	28.72 +/- 6 (20-60)	NA
Northern Africa	**Sudan**	**Taher Gorish et al., 2019**	No confirmation	BD	ELISA	None	2019	1% (4/394)	Med. 33 (18-53)	91,60%
Northern Africa	**Sudan**	**Taher Gorish et al., 2019**	Other population (Patients)	Patients	ELISA	PCR	2019	0% (0/106 hematologic malignancies)	41±12.4	67,90%
Northern Africa	**Tunisia**	**Saxinger et al., 1984**	Other population (Patients)	Patients	ELISA			9% (2/22 malignant lymphoma patients); 2% (6/256 mammary carcinoma patients)	NA	NA
Northern Africa	**Tunisia**	**Larouze et al., 1985**		PW	RIPA	None		0% (0/442)	17-47	0%
Northern Africa	**Tunisia**	**Moojat et al., 1999**		BD + else	ELISA	WB		0% (0/500 BD); 0% (0/387 multitransfused patients; 0.4% (1/232 hemodialysed patients); 0% (0/25 HIV+)	(18-65) Med. 38	NA
Western Africa	**Benin**	**Dumas et al., 1991**		Pop + BD	EIA + IFA	WB (NA) + RIPA	1988-89	1.5% (39/2625); 0% (0/1,300 BD)	NA	49%
Western Africa	**Benin**	**Houinato et al., 1996**		Pop	EIA + IFA	WB (NA) + RIPA	1990	1.9% (31/1671)	NA	49%
Western Africa	**Benin**	**Houinato et al., 2002**		Pop	EIA	WB (2.3) + RIPA	1998	4.5% (80/1760 rural pop); 5.3% (31/580 urban pop)	40 +/-16 (15-87)	53%
Western Africa	**Burkina Faso**	**De thé et al., 1985**	No confirmation	NA	ELISA x2	None		4.7% (2/43)	20-49	NA
Western Africa	**Burkina Faso**	**Collenberg et al., 2006**		PW + BD	EIA	WB (2.4)		1%*(5/191 BD); 0.8%*(2/492 PW)	BD: 26 (14-48) PW: 25 (16-45).	BD: 92%; PW: 0%
Western Africa	**Burkina Faso**	**Nébié et al., 2007**	No confirmation	BD	ELISA	None		3.1%* (11/355)	28 ± 7.9 (16-57)	71%
Western Africa	**Burkina Faso**	**Ouedraogo et al., 2018**	No confirmation	MSM	ELISA	None	2013	4% (13?/329 MSM)	22.9 ± 4.0	100%
Western Africa	**Burkina Faso**	**Ouedraogo et al., 2022**	No confirmation	FSW	ELISA	None	2013	11.8%* (NA/348)	18	0%
Western Africa	**Côte d'Ivoire**	**De thé et al., 1985**	No confirmation	NA	ELISA x2	None	1970s	16% (16/100)	(0-50+)	NA
Western Africa	**Côte d'Ivoire**	**Denis et al., 1988**		PW	IFA	WB	1986-1987	1.8% (15/814)	(<20-40+)	0%
Western Africa	**Côte d'Ivoire**	**De thé et al., 1989**	Patients	TSP/HAM	ELISA	WB (DPN)	1986/88	10% - 3% (7 - 4/68 + 240)	NA	NA
Western Africa	**Côte d'Ivoire**	**Ouattara et al., 1989**		Pop + BD	ELISA	WB (DPN)	1987	1.1% (15/1,334 pop); 1.7% (7/414 BD)	pop: 26 (1-81); BD: 28 (15-60)	pop: 55% BD: 79%
Western Africa	**Côte d'Ivoire**	**Verdier et al., 1989**		Pop + PW	IFA	WB (home- made)	1986-1987	7.4% (29/390 SW);1.9% (10/513 PW);0.6% (1/155 Students); 2.2% (7/312 adults); 1.6%(5/311 male hostel staff) ;	students 23; adults 32; male hostel staff 33; PW : 25	students 4.2; adults 0.9; male hostel staff 1; PW :0
Western Africa	**Côte d'Ivoire**	**Mossoun et al., 2017**		Rural pop	ELISA	WB (2.4) + PCR	2011-2013	0.7% (4/574 rural pop)	39,7	39,4%
Western Africa	**Ghana**	**Biggar et al., 1984**	No confirmation	Rural + Urban pop	ELISA + competitive ELISA	None	1978	5.8%(9/153 adults); 2.7% (10/364 children)	(6-11); (11-30+)	adults: 10%, children 40%
Western Africa	**Ghana**	**Saxinger et al., 1984**	*No confirmation*		ELISA			8.1% (19/236)		

Western Africa	**Ghana**	**Biggar et al., 1993**		Rural pop + PW + patients + FSW	ELISA	Immunoblot + PCR (for a subset)	1989-90	1.4% (32/2,262 rural pop); 2% (22/1,120 PW); 1% (1/116 FSW)	40%	NA
Western Africa	**Ghana**	**Goubau et al., 1993**	Other population (Refugees)	Refugees	ELISA	WB + IFA		0.9% (2/230)	NA	NA
Western Africa	**Ghana**	**Aidoo et al., 1994**	No confirmation	AIDS	PA	None		5.5% (NA/255 AIDS patients)		
Western Africa	**Ghana**	**Hishida et al., 1994**	Other population (HIV patients)	AIDS	IFA + PA	WB (Fujirebio)	1990-92	4.8% (14/290 suspected AIDS patients)	>15	NA
Western Africa	**Ghana**	**Lal et al., 1994**	Other population (Patients)	Patients		WB (2.3)		33.1% (46/139)		
Western Africa	**Ghana**	**Brandulf et al., 1999**	Other population (HIV patients)	HIV	PA	None		6.6% (12/182 HIV); 0% (0/88 patients HIV negative)	34 (23-45)	56%
Western Africa	**Ghana**	**Sarkodie et al., 2001**	No confirmation	BD	EIA x2	None		0.4% overall (17/3807 BD)<br>0.6% replacement BD(1) ; 0.4% volontary BD (2)	(1) Med. 18 (16-52);<br>(2) Med. 33 (16-56)	(1) 62%<br>(2)90%
Western Africa	**Ghana**	**Ampofo et al., 2002**	No confirmation	BD	PA	None	1999	0.7% (6/808)	16-55	94%
Western Africa	**Ghana**	**Adjei et al., 2003a**	No confirmation	BD	PA	None	2001	4.2% (11/265)		83%
Western Africa	**Ghana**	**Adjei et al., 2003b**	Other population (HIV patients)	AIDS	PA	None		11.3% (14/124)	16-54	68%
Western Africa	**Ghana**	**Apea-Kubi et al., 2006**	No confirmation	Women	PA	None	2000-2001	2.7%(8/294 PW); 2.7%(6/223 gynaecological patients)	29 ± 6.9 (16-41+)	0%
Western Africa	**Ghana**	**Armah et al., 2006**		PW	PA	WB (2.4)		2.1% (20/960)	25.6 ± 5.8 (15-41)	0%
Western Africa	**Ghana**	**de Mendoza et al., 2019**		Patients	EIA	INNO-LIA	2015	1.3% (4/305)	Med. 26 (IQ 18-35)	32%
Western Africa	**Guinea**	**Gessain et al., 1993**		BD	ELISA x2	WB (Ortho Diag.)	NA	1.2% (22/1785)	<40	93%
Western Africa	**Guinea**	**Jeannel et al., 1995**		Rural pop	ELISA	WB (2.2)	1992	1.1% (24/2285)	23 (2-97)	50%
Western Africa	**Guinea-Bissau**	**Lillo et al., 1991**	Other population (HIV patients)	HIV2	ELISA + EIA	WB (Genova Lab)	NA	9.9% (73/735)	NA	NA
Western Africa	**Guinea-Bissau**	**Nauclér et al., 1992**		PW + Urban pop	ELISA	WB (DPN + Diag. Biotechnology)	NA	6.4% (63/687 patients); 3.7% (19/512 male police officers); 3.3% (9/ 272PW)	patients (15-60+)<br>others : NA	patients: 56%; PO: 100%; PW: 0%
Western Africa	**Guinea-Bissau**	**Norrgren et al., 1995**		Urban pop	ELISA	WB (2.2)	90-92	4% (55/1384 police officers)	15-55+	90%
Western Africa	**Guinea-Bissau**	**Andersson et al., 1997**		PW	ELISA	WB (2.4)	1993	2.2% (27/1231)	NA	0%
Western Africa	**Guinea-Bissau**	**Melbye et al., 1998**	No confirmation	Old people	IFA	None	1989	9% (31/346)	50-70+	44%
Western Africa	**Guinea-Bissau**	**Larsen et al., 2000**		Urban pop	EIA/ELISA	WB (2.3)	1995-96	3.6% (76/2127)	30.7-32.1 (15-45+)	44%
Western Africa	**Guinea-Bissau**	**Holmgren et al., 2002**		Rural pop	ELISA x2	WB (2.4) + PCR	1989-91	5.2% (130/2501)	40.6 (15-99)	36%
Western Africa	**Guinea-Bissau**	**Ariyoshi et al., 2003**	Other population (HIV patients)	HIV-2	ELISA	PCR (+/- WB)	1991	26%(33/126 HIV-2)<br>7% (15/159 controls HIV-2 negative)	44-52 (range NA)	31% HIV-2, 27% controls
Western Africa	**Guinea-Bissau**	**Holmgren et al., 2003**		Rural pop (old)	ELISA	WB (2.4)	1998-2000	7.1% (112/1581)	57 (35-91)	48%
Western Africa	**Guinea-Bissau**	**Norrgren et al., 2008**	Duplicate population	TB patients + pop	ELISA	WB (2.3)	1994-97	11.4% (32/280 TB patients);<br>3.5% (74/2,117 healthy controls)	TB: 38.2 ± 15.8;<br>controls: 31.3 ± 13.2	TB: 60%<br>controls: 44.5%
Western Africa	**Guinea-Bissau**	**Zehender et al., 2008**		PW	ELISA	PCR		2.6% (11/427 PW)	23.2 ± 5.2	0%
Western Africa	**Guinea-Bissau**	**da Silva et al., 2009**		Urban pop	ELISA	INNO-LIA	2004-07	0% (NA/2547)	Med. 28	40%
Western Africa	**Guinea-Bissau**	**Mansson et al., 2010**	Other population	STDs?	ELISA	INNO-LIA	2006-08	2.8% (19/671)	24.5 (14-48)	0%
Western Africa	**Guinea-Bissau**	**Van Tienen et al., 2010**		Rural pop	ELISA x2	PCR	1990,1997,2 007	0% (/2895) 5.2% (1990); 5.9% (1997); 4.6% (2007)	Med. 31 (15-65+)	40%
Western Africa	**Guinea-Bissau**	**Van Tienen et al., 2012**	Low numbers	Mothers & children	ELISA x2	PCR	2004	25% (14/55 children with HTLV-1 infected mother); 0% (0/70 children with HTLV-1 negative mothers)	Mothers Med. 34;<br>Children <15	0%; NA
Western Africa	**Guinea-Bissau**	**Kjerulff et al., 2018**	Duplicate population	Urban pop	CLEIA	PCR (+/- INNO-LIA)	2014-16	0% (/2583)		
Western Africa	**Guinea-Bissau**	**Jensen et al., 2019**		Urban pop	CLEIA	PCR (+/- INNO-LIA)	2014-16	2.7% (71/2583)	33,1	42%

Western Africa	**Guinea-Bissau**	**Hønge et al., 2020**	Duplicate population	Urban pop	CLEIA	PCR (+/- INNO-LIA)	2014-16	2.7% (74/2715)		
Western Africa	**Liberia**	**Hunsmann et al., 1984**		Rural pop	ELISA	RIPA	NA	1.6% (10/620)	NA	NA
Western Africa	**Mali**	**Larouze et al., 1985**	Low numbers	Immigrants	RIPA	None		0% (0/69)	19-40	100%
Western Africa	**Mali**	**Denis et al., 1988**	Low numbers	PW	IFA	WB	1986-1987	0% (0/63)	(<20-40+)	0%
Western Africa	**Mali**	**Diarra et al., 2014**	No confirmation	BD	ELISA x2	None	2011	1.4% (11/799 BD); 6.4% (10/156 multi-transfused patients)	Med. 26 (18-60)	87%
Western Africa	**Niger**	**Denis et al., 1988**	Low numbers	PW	IFA	WB	1986-1987	1.6% (1/61)	(<20-40+)	0%
Western Africa	**Nigeria**	**Flemming et al., 1983**	Duplicate population	BD	IFA	None	NA	3.7% (6/161 BD); 44%(4/9 CLL patients); 20%(1/5 NHL), 12.5% (1/8 Burkitt's lymphoma)	NA	NA
Western Africa	**Nigeria**	**Hunsmann et al., 1984**		BD	ELISA	RIPA		2.6% (10/390 BD, including 161 from Flemminet el al.,1983)	NA	NA
Western Africa	**Nigeria**	**Saxinger et al., 1984**	Other population (Patients)	Patients	ELISA	None	NA	22% (2/9 T-cell lymphoma)		
Western Africa	**Nigeria**	**Flemming et al., 1986**		BD	ELISA	RIPA		2% (15/736 BD); 5% (1/20 relatives of HTLV-1+); 9% (9/100 lymphoma patients (9%)	NA	>95%
Western Africa	**Nigeria**	**Okpara et al., 1988**		BD	ELISA	RIPA		0.8% (2/301)	(19-50)	98%
Western Africa	**Nigeria**	**Olusanya et al., 1990**		Urban pop	EIA	WB (DPN)	1987-1988	0.5% (2/385)	(15-42)	19.5%
Western Africa	**Nigeria**	**Dada et al., 1993**	Other population (SW)	FSW	ELISA	WB	1990/91	2.7% (20/752)	Med. 25 (12-50)	0%
Western Africa	**Nigeria**	**Olaleye et al., 1993**	Duplicate population	Diverse	NA			NA (NA/4153)	NA	NA
Western Africa	**Nigeria**	**Williams et al., 1993**	Low numbers	BD + children + ATL	ELISA	*WB*		7% (9/123 BD); 6.5% (3/46 children); 20% (6/30 NHL patients)	BD: 23.9; children: 8.9 Patients: NA	NA
Western Africa	**Nigeria**	**Olaleye et al., 1994**		Rural pop + patients + FSW + STDs	EIA x3	WB	1985-91	1.8%(1,640 survey participants), 6.4%(140 TB patients); 3.2% (1,285 patients); 8.3% (60 FSW); 3.3% (152 HCW); 16.3% (876 STIs patients)		
Western Africa	**Nigeria**	**Olaleye et al., 1995**		PW	EIA x2	WB (home-made)	1991	5.5% (20/364)	(17-42)	0%
Western Africa	**Nigeria**	**Olaleye et al., 1996**	Other population (Patients)	Patients + STDs		PCR		1.5%(1/65 lymphoma), 7.5% (3/40 STIs patients), 4% (1/25 candidiasis patients), 3% (2/60 healthy individuals)		
Western Africa	**Nigeria**	**Olumide et al., 1996**	Other population	STDs	ELISA	WB	1992-94	7.2% (25/348)	(20-45)	
Western Africa	**Nigeria**	**Analo et al., 1998**	No confirmation	BD	ELISA	None		0.7% (3/406)	NA	NA
Western Africa	**Nigeria**	**Olaleye et al., 1999**		mother/PW? & children	ELISA x2 + EIA	WB (home-made)	1993	3.3%(15/460 mothers); 1.1% (5/476 children)	NA	0%
Western Africa	**Nigeria**	**Eltom et al., 2003**	Duplicate population	Pop + patients + SW + STDs	ELISA	WB	1992-94	Women: 2%(6/287 PW); 2.6% (3/114 STDs); 3.2% (28/863 FSW) ; Men : 3.2% (16/479 pop); 5.7% (15/259 STDs)		
Western Africa	**Nigeria**	**Forbi et al., 2007**	No confirmation	Diverse	ELISA	None	NA	5.1% (4/78 students); 16.7% (20 /120 PW); 22.9%(38/166 FSW)	students: 13; PW: 26; FSW: 23	0%
Western Africa	**Nigeria**	**Terry et al., 2011**		BD	ELISA	WB (2.4)	2008	1.9% (7/372)	(18-62)	89%
Western Africa	**Nigeria**	**Akinbami et al., 2014**	Other population (Patients)	Patients	ELISA	WB (2.4)		5.1% (39 solid malignancies and 15 leukemia patients);	51.9 ±17.8	58%
Western Africa	**Nigeria**	**Durojaiye et al., 2014**		BD	ELISA	WB (2.4)		0.5% (1/210)	33 ± 8.9 (<20-59)	87.6%
Western Africa	**Nigeria**	**Okoye et al., 2014**	No confirmation	PW	ELISA	Electroblotting	2010	0.5% (1/200)	28.9 ± 4.17 (16-40)	0%
Western Africa	**Nigeria**	**Iyalla et al., 2015**	No confirmation	BD + PW	ELISA	None		1.4% (2/139 BD); 0% (0/221 PW)	BD: 18-64 Pregnant: NA	BD: 88%
Western Africa	**Nigeria**	**Nasir et al., 2015**	Other population (HIV patients)	HIV	ELISA	PCR		6.5% (12/184 HIV+)		
Western Africa	**Nigeria**	**Okoye et al., 2015**	No confirmation	BD	ELISA	Electroblotting	2010	0% (0/300)	26.85 ± 8.52 (16-55)	96%

Western Africa	**Nigeria**	**Oladipo et al., 2015**	No confirmation	BD	ELISA	None	2013	25.8% (24/93)	45 +/- 2.3	68%
Western Africa	**Nigeria**	**Ma'an et al., 2016**		BD	ELISA	None	NA	0% (0/500)	29.9 +/- 8.9 (18-59)	80,6%
Western Africa	**Nigeria**	**Manga et al., 2016**		BD	ELISA	WB (2.4)	NA	0% (0/355)	30.77 +/-8.27 (range 18-58)	99,2%
Western Africa	**Nigeria**	**Opaleye et al., 2016**	No confirmation	PW	ELISA	None	NA	24.2% (44/182)	(15-49)	0%
Western Africa	**Nigeria**	**Udeze et al., 2018**	No confirmation	PW	ELISA	None	2017	1.1% (3/276)	28.2 (16-40)	0%
Western Africa	**Nigeria**	**Anyanwu et al., 2019**	Low numbers	BD +Patients + HIV	ELISA	PCR		0% (0/200 BD; Patients + HIV))	35.28±13.6 (6-71)	60%
Western Africa	**Nigeria**	**Hananiya et al., 2019**	No confirmation	PW	ELISA	None	2017	3.2% (3/190)	(15-45)	0%
Western Africa	**Nigeria**	**Dangana et al., 2021**	No confirmation	PW	ELISA	None		10.3% (16/156)		
Western Africa	**Senegal**	**Hunsmann et al., 1984**		Rural pop	ELISA	RIPA		1.2% (12/993)	NA	NA
Western Africa	**Senegal**	**Larouze et al., 1985**		PW	-	RIPA		0.2% (1/415)	(16-50)	0%
Western Africa	**Senegal**	**Denis et al., 1988**		PW	IFA	WB	1986-1987	0.4% (1/281)	(<20-40+)	0%
Western Africa	**Senegal**	**Kaplan et al., 1994**	Other population (TB patients)	TB patients	ELISA	WB + RIPA	1991	1.5% (3/197 TB patients); 1.1% (2/181 controls)	30 (17-70)	64,50%
Western Africa	**Senegal**	**Diop et al., 2006**		BD	ELISA	WB (2.4)	2002	0.2% (8/4,900 BD)	29.6 (18-65)	73%
Western Africa	**Sierra Leone**	**Ronday et al., 1996**	Other population (Patients)	Patients	EIA	WB (NA)	1992	6%(6/93 uveitis patients); 5% (5/100 other patients)	37 (12-70)	F:M ratio 1:0.7-0.8
Western Africa	**Sierra Leone**	**Yendewa et al., 2019**	Other population (HIV patients)	HIV	CLEIA	None		0% (0/211)	Med. 36 (IQR 32-44)	36,50%
Western Africa	**Sierra Leone**	**Yendewa et al., 2021**	Other population (HIV PW and children)	HIV PW + children	CLEIA	None	2019	0% (0/88 children); 4.3% (2/47 teenagers); 4.2% (2/48 PW)		
Western Africa	**The Gambia**	**Pepin et al., 1991**	Other population (SW)	SW	ELISA + PA	WB (DPN)	1988-89	10.4% (37/355 SW)		
Western Africa	**The Gambia**	**Del Mistro et al., 1994**		"PW"? & children	ELISA	WB (DPN)	1988-89	1.2%(11 /909 mothers); 0.1%(1/916 children)	Mother (14-47). Children (12 -17 months)	Mother : 0% Children: NA
Western Africa	**Togo**	**Denis et al., 1988**		PW	IFA	WB	1986-1987	1.2% (7/565)	(<20-40+)	0%
Western Africa	**Togo**	**Balogou et al., 2000**		Rural pop + Patients	ELISA	WB (DPN)	1987, 1990	1.2% (21/1,717 pop);<br>1.8%(15/828 neurological patients)<br>1.6% (4/244 other patients)	34 +/-20 (15-60+);<br>41.8 +/-9.7 (15-90);<br>40.8 +/- 8.8 (16-90)	45%;<br>57%;<br>51%
Central Africa	**Cameroon**	**Delaporte et al., 1989**		Rural pop	ELISA	WB	1987-1988	0.5-4.2-11.8% (NA/853)	(15-44)	NA
Central Africa	**Cameroon**	**Ndumbe et al., 1992**		BD+PW+Patients+rural +STDs	ELISA + PA	WB (Ortho Diag.)	1987	2.7% (4/150 villagers); 2.4%(5/210 patients); 0.6% (1/170 PW); 0% (0/100BD); 0% (0/151 STIs patients)*	(18-45)	NA
Central Africa	**Cameroon**	**Froment et al., 1993**		Rural pop	ELISA	WB	1984	1% (3/301)	(1-70)	NA
Central Africa	**Cameroon**	**Goubau et al., 1993**		Rural pop (baka)	ELISA	WB	1967-1971	0.9% (2/214 Pygmy "Baka")	NA	NA
Central Africa	**Cameroon**	**Mauclere et al., 1993**	Other population (SW)	SW	EIA + IFA	WB (2.3)	1992	0.3% (1/391)		0%
Central Africa	**Cameroon**	**Ndumbe et al., 1993**		Rural pop (baka)	ELISA	WB	1987	10.9% (15/138 Pygmy "Baka")	(18-45)	NA
Central Africa	**Cameroon**	**Kowo et al., 1995**		Rural pop	PA	WB (2.3)	1994	0% (0/168)	34 (12-80)	48%
Central Africa	**Cameroon**	**Mauclere et al., 1995**	Other population (SW)	SW	ELISA + IFA	WB (2.3)	1993	0.9% (3/332)		0%
Central Africa	**Cameroon**	**Mauclere et al., 1997**		Rural pop	ELISA + IFA	WB (2.3)	1992-1994	1.1% (42/3783)		48%
Central Africa	**Cameroon**	**Mboudjeka et al., 1997**		Rural pop	PA	WB (Furijebio)	1995	3.4% (3/87 Pygmies "Baka"; 1.2% (3/259 Bantus)	NA	NA
Central Africa	**Cameroon**	**Machuca et al., 2005**		Rural pop	EIA	WB (2.4)	NA	6.6% (49/747)	NA	NA
Central Africa	**Cameroon**	**Filippone et al., 2012**		Rural pop	ELISA	WB (2.4)	*NA*	1.9% (38/1,968)	44 (5-90)	50,3%
Central Africa	**Cameroon**	**Ramassamy et al., 2022**		Rural pop	ELISA	WB (2.4) + PCR	2018-2021	0.7% (23/3,400)	40 (15-90)	46%
Central Africa	**Central African Republic**	**Larouze et al., 1985**	Other population (Patients)	Patients (NA)	RIPA	None	NA	0% (0/77)	16-60	Sex ratio 1.02
Central Africa	**Central African Republic**	**Gessain et al., 1992**		Rural pop	ELISA	WB (2.3)	1990-1992	1.2% (5/410 Pygmies "Baka"); 0% (0/279 Bantus)	NA	NA

Central Africa	**Central African Republic**	**Goubau et al., 1993**		Rural pop (baka)	ELISA	WB	1967-1971	0% (0/504 Pygmies "Baka")	NA	NA
Central Africa	**Central African Republic**	**Pépin et al., 2010**		Rural pop	ELISA + EIA	INNO-LIA + PCR	2006	7.4% (67/903)	Med. 61-64 (57-74)	37-55%
Central Africa	**Chad**	**Delaporte et al., 1989**		Rural pop	ELISA	WB	1987-1988	0.5-2% (NA/666)	(15-44)	NA
Central Africa	**Chad**	**Louis et al., 1990**	No data (no abstract)	Pop			1989	0-1.6% (NA/?)	(15-44)	
Central Africa	**Congo**	**Larouze et al., 1985**	Mixed population	*BD + Pop*	RIPA		NA	0.6% (2/360)	(10-70)	sex ratio 1.30
Central Africa	**Congo**	**De thé et al., 1989**	Patients	TSP/HAM	ELISA	WB (DPN)	1985	0% (0/62)	NA	NA
Central Africa	**Congo**	**Tuppin et al., 1996**		PW	EIA	WB (2.3)	1992	0.7% (14/2070)	25	0%
Central Africa	**DR Congo (Ex Zaire)**	**Hunsmann et al., 1984**	Low numbers	Rural pop	ELISA	RIPA		6.5% (4/62)	NA	NA
Central Africa	**DR Congo (Ex Zaire)**	**Biggar et al., 1985**	No confirmation	Rural pop	ELISA	None	1984	14% (35/250)	32 (8-78)	44%
Central Africa	**DR Congo (Ex Zaire)**	**De thé et al., 1985**	No confirmation	NA	ELISA x2	None		14.4% (27/187)	(0-50+)	NA
Central Africa	**DR Congo (Ex Zaire)**	**Gazzolo et al., 1985**	Low numbers	PW + Pop: out-patients + TB	ELISA + competitive ELISA	WB (home- made)		13% (24/182)	(5-71)	46%
Central Africa	**DR Congo (Ex Zaire)**	**Gazzow et al., 1985**	No data (no abstract)	Diverse				0% (/)		
Central Africa	**DR Congo (Ex Zaire)**	**Getchell et al., 1987**	Other population (HIV patients)	AIDS	IFA	WB (DPN)		0% (1/1)	45	1
Central Africa	**DR Congo (Ex Zaire)**	**De thé et al., 1989**	Other population (Patients)	Patients	ELISA	WB (DPN)	1985 +87	0% (0/35)	NA	NA
Central Africa	**DR Congo (Ex Zaire)**	**Goubau et al., 1990**	Other population (Patients)	Patients + workers	ELISA + IFA	WB		14% (6/42 HCW) + 13.9% (22/158 patients)		
Central Africa	**DR Congo (Ex Zaire)**	**Wiktor et al., 1990**	Low numbers	SW + PW + Hospi	ELISA	WB	1986	2% (3/153 PW); 9.7% (12/124 SW);3.2%(12/377 Kinshasa SW); 5.5%(4/73 patients)		
Central Africa	**DR Congo (Ex Zaire)**	**Wiktor et al., 1990**	same article (other pop)	SW	ELISA	WB	NA			
Central Africa	**DR Congo (Ex Zaire)**	**Goubau et al., 1992**	Low numbers	Rural pop	ELISA	WB	1991	1.3% (2/158)	NA (adults)	69%
Central Africa	**DR Congo (Ex Zaire)**	**Goubau et al., 1993**	Low numbers	Rural pop (baka)	ELISA	WB	1967-1971	1% (1/102 Pygmies "Baka")	NA	NA
Central Africa	**DR Congo (Ex Zaire)**	**Goubau et al., 1993**		BD + PW + mothers + urban + rural + patients	ELISA + IFA	WB	NA	4% (21/530 BD) + 4.6%(19/414 PW); 1.2% (17/1409 mothers); + SW ; 3.2%(2/62 urban population); 1% (4/385 rural population); 3% (22/641 neurological patients or suspected of AIDS)		
Central Africa	**DR Congo (Ex Zaire)**	**Jeannel et al., 1993**		Rural pop + children + patients	ELISA + PA	WB	1990	4.4% (32/725 pop); 0% (0/437 children)	20 +/- 19	46%
Central Africa	**DR Congo (Ex Zaire)**	**Dube et al., 1994**	Other population (Patients)	Leprosy patients	ELISA	WB + PCR	1969	26.8% (57/213 Leprosy patients); 45%(17/37 healthy donors)	NA	NA
Central Africa	**DR Congo (Ex Zaire)**	**Garin et al., 1994**	Other population (Patients)	Patients	ELISA	WB (2.2) + PCR	1990+91	35.7% (35/98 neurological patients)	34 (1-100)	46%
Central Africa	**DR Congo (Ex Zaire)**	**Delaporte et al., 1995**		PW + SW	ELISA	WB	1990	3.7%(43/1,160 PW); 7.3% (86/1,183 SW)	NA	0%
Central Africa	**DR Congo (Ex Zaire)**	**Tylleskar et al., 1996**	Low numbers	Rural pop	ELISA	WB	NA	0% (0/132: 23 konzo cases ; 109 healthy villagers)	Med. 30 (4-68)	49%
Central Africa	**DR Congo (Ex Zaire)**	**Lechat et al., 1997**	Other population (Patients)	Leprosy patients	ELISA	WB (DPN)	1969	37.4%(141/377 Leprosy patients); 25.2% (36/143 controls)		54%

Central Africa	**DR Congo (Ex Zaire)**	**Hogan et al., 2016**		Urban pop	EIA	WB (2.4)	2012	3.1% (26/839)	Med. 75 (70-97)	NA
Central Africa	**DR Congo (Ex Zaire)**	**Mossoun et al., 2017**		Rural pop	ELISA	WB (2.4) + PCR	2011-2013	1.3% (4/302)	40,4	43%
Central Africa	**DR Congo (Ex Zaire)**	**Halbrook et al., 2021**		Rural pop	EIA	WB (2.4)		5% (152/3,051)	(0-99)	43%
Central Africa	**Equatorial Guinea**	**Delaporte et al., 1989**		Rural pop	ELISA	WB (DPN)	1987-1988	5.6% / 10.1% (NA/792)	(15-44)	NA
Central Africa	**Gabon**	**Hunsmann et al., 1984**		Rural pop	ELISA	RIPA	81-93	2.7% (16/585)	NA	NA
Central Africa	**Gabon**	**Delaporte et al., 1988**	same article	Children	ELISA	WB (DPN)	86-87	2-2.4% (NA/684)	(1-14)	
Central Africa	**Gabon**	**Delaporte et al., 1988**		Rural + Urban pop	ELISA	WB (DPN)	86-87	5% (NA/759 urban pop); 8.6-10.5% (NA/1,115 rural pop)	(15-54)	
Central Africa	**Gabon**	**Delaporte et al., 1989**	Duplicate population	Rural pop	ELISA	WB (DPN)	86-87	9.2% (82/885)		
Central Africa	**Gabon**	**Delaporte et al., 1991**	Duplicate population	Rural pop	ELISA	WB + PCR	86-87	0% (/322)		
Central Africa	**Gabon**	**Schrijvers et al., 1991**		PW	ELISA	WB (DPN)	1986-87	6.8% (44/651)	(15-54)	0%
Central Africa	**Gabon**	**Berteau et al., 1993**		PW + BD	ELISA	WB (DPN)	1989-1990	6% (42/704 BD); 5.5% (35/633 PW)	BD: (16-60); PW: (13-49)	BD 76%; PW: 0%
Central Africa	**Gabon**	**Delaporte et al., 1993**	Other population (Children)	Children	ELISA	WB (DPN)	86-87	2.8% (17/610 children)	(0.5-14)	
Central Africa	**Gabon**	**Berteau et al., 1994**	Other population (Children)	Children	ELISA	WB		4.7% (32/680)	(0-5)	54%
Central Africa	**Gabon**	**Le Hesran et al., 1994**		Rural pop	ELISA	WB (Ortho Diag.)	1988	8.5% (106/1,240)	26±18	43%
Central Africa	**Gabon**	**Bertherat et al., 1998**		Urban pop	ELISA	WB (2.4)		6.6% (30/456)	(14-55)	
Central Africa	**Gabon**	**Moynet et al., 2001**		Rural pop	ELISA	WB	1992	5.3% (NA/604)	NA	ratio W/M 1.06
Central Africa	**Gabon**	**Etenna et al., 2008**		PW	ELISA	WB (2.4)	2008	2.1% (19/907)	(14-40)	0%
Central Africa	**Gabon**	**Kazanji et al., 2015**	Low numbers	Rural pop	ELISA x2	WB (2.4)	2015	0% (7/78)		
Central Africa	**Gabon**	**Moukandja et al., 2017**	No confirmation	PW	ELISA	None	2017	2.8% (NA/973)	25.84 ± 6.9 (14-45)	0%
Central Africa	**Gabon**	**Caron et al., 2018**		Rural pop	ELISA	WB (2.4)	2005-2008	7.3% (320/4,381)	47±14 (15-90)	ratio 0.9
Central Africa	**Gabon**	**Djuicy et al., 2018**		Rural pop	ELISA	WB (2.4) + PCR	2018	8.7% (179/2,060)	Med. 48 (15-100)	58%
Central Africa	**Gabon**	**Ramassamy et al., 2020**		BD	ELISA	WB (2.4) + PCR	2020	0.7% (23/3,123)	31 (17-59)	82%
Eastern Africa	**Burundi**	**Bonis et al., 1994**		Hospi + Pop	EIA	WB (2.3)	1993	0.4%(2/485 hospitalized patients); 1.34% (7/519 pop)	35.3 ± 13.4	51%
Eastern Africa	**Djibouti**	**Fox et al., 1989**	Other population (SW)	SW client	NA		NA	0% (0/105)		100%
Eastern Africa	**Djibouti**	**Fox et al., 1989**	Other population	SW + STDs	PA	WB (DPN)	1988	1.2% (4/327 SW); 0% (0/240 males with STD)	26	0%
Eastern Africa	**Eritrea**	**Andersson et al., 1999**		PW+SW+Children+else	ELISA	WB (2.4)		0% (0/589: 97 SW; 113 PW, 91 workers; 65 guerilla; 161 children; 62 Rashaidas ethnicity)		
Eastern Africa	**Ethiopia**	**Teckle-Haimanot et al., 1991**		Patients + BD + else	EIA + IFA	WB + RIPA	1988-1989	0% (250 Leprosyp atients; 201 dermatological patients ; 47 HCW; 140 BD; 113 urban outpatients; 112 rural pop)	(0-59)	controls 66%
Eastern Africa	**Ethiopia**	**Goubau et al., 1993**	Low numbers	Rural pop (baka)	ELISA	WB	1967-1971	4.8% (2/42 Pygmies Baka)	NA	NA
Eastern Africa	**Ethiopia**	**Vrielink et al., 1995**		BD + patients + STDs	ELISA	WB (2.3)	<1994	0.19% (3/1,600 BD); 0.6% (6/933 other population: urban, rural pop, STIs patients).	NA	NA
Eastern Africa	**Ethiopia**	**Ramos et al., 2011**	No confirmation	Rural patients	CLEIA	None	2010	0% (0/556 rural outpatients)	NA	41%
Eastern Africa	**Kenya**	**Hunsmann et al., 1984**	Other population	Foreigner students	ELISA	RIPA		1.7% (4/231)	NA	NA
Eastern Africa	**Kenya**	**Biggar et al., 1985**	No confirmation	Rural pop + patients	ELISA	WB on a subset	1980-84	30% /(592 + 100/592 volonteers + 36% 100 parasitic patients)		
Eastern Africa	**Kenya**	**Songok et al., 1988**	Other population (HIV patients)	AIDS	ELISA	WB + PA	NA	0.4% (4/913)		

Eastern Africa	**Kenya**	**He et al., 2016**	Other population (HIV patients)	Women HIV+/HIV neg	None	PCR	NA	3.6% (4/111 HIV negative); 19.5% (22/113 HIV positive) DNA extraction on cervical smears (or cercival carcinoma biopsy	Med. 35 (21-52)	0%
Eastern Africa	**Madagascar**	**Larouze et al., 1985**		BD	-	RIPA		0% (0/198)	(38-61)	sex ratio M/F 3.37
Eastern Africa	**Madagascar**	**Cnudde et al., 1991**	Other population (Patients)	Patients	ELISA	WB (DPN)	1988	0% (0/101+7)	NA	NA
Eastern Africa	**Malawi**	**Candotti et al., 2001**	No confirmation	BD	EIA	None	NA	2.5% (4/159)	NA	NA
Eastern Africa	**Malawi**	**Fox et al., 2016**		Mothers "PW?" & children	ELISA	WB (2.4) + PCR	2006-10	1% / 1% (4/5/418 mothers / 534 children)		
Eastern Africa	**Mozambique**	**Van Rensburg et al., 1995**	Other population	Refugees	NA		NA	5.4% (/398)		
Eastern Africa	**Mozambique**	**Melo et al., 2000**		PW	ELISA	WB (2.4)	1997	0.7% / 2.3% (1 / 4/132 pregnant + 171 STD patients)	23.3 ± 5.6<br>24.7 ± 6.5	0%
Eastern Africa	**Mozambique**	**Cunha et al., 2007**		BD	ELISA	WB (2.4) + PCR	2004	1.1% (18/1578)	32.8 ± 11.7 (15–82)	78.1%
Eastern Africa	**Mozambique**	**Bhatt et al., 2009**	Other population (HIV patients)	HIV	EIA	WB (2.4)		4.5% (32/704)	NA	NA
Eastern Africa	**Mozambique**	**Gudo et al., 2009**		BD	EIA x3	WB +/- PCR	2006	0.9% (18/2019)	46.57% 16-29<br>24.82% 30-39<br>28.60% >40	81.2%
Eastern Africa	**Mozambique**	**Caterino-de-Araujo et al., 2010**		Patients	EIA	WB (2.4)		2% (15/752)		
Eastern Africa	**Mozambique**	**Augusto et al., 2017**	Other population (HIV patients)	HIV	ELISA + EIA	None		1.6% (8/515)	Med. 29 (IQR 25-33)	86.4%
Eastern Africa	**Mozambique**	**Manhiça et al., 2017**	Other population (HIV children)	Children HIV+	ELISA	PCR	2010-11	3.9% (37/945)	Med. 6 (IQR 4-9)	50.6%
Eastern Africa	**Rwanda**	**Rwandan HIV study group., 1989**		Rural + Urban pop	EIA	WB (DPN)	1986	0.2% (5/2612)	(0-40+)	51%
Eastern Africa	**Rwanda**	**Van de Perre et al., 1989**	Other population (HIV patients)	HIV				0% (0/20 adults + 12 children)		
Eastern Africa	**Seychelles**	**Lavanchy et al., 1991**		Rural + Urban pop	EIA	RIPA		6.2% (65/1055)	(25-64)	47%
Eastern Africa	**Seychelles**	**Aubry et al., 2013**	No numbers	BD	ELISA		2008-2012	*1.9%* (14/*750* estimated BD)		
Eastern Africa	**Somalia**	**Scott et al., 1991**		Patients + SW + Leprosy + else Pop (outpatients) + STDs	EIA	WB (DPN) + RIPA	1989	0% (57 SW; 235 STDs patients; 22 leprosy patients); 0.09%(1/1,133 oupatients or immigrants); 29 infectious disease, 0/22 leprosy, 51+71+115 TB; 145 visitors; 440 rehab camp; 125 ethiopian immigrants	30.6 +/- 13.8	
Eastern Africa	**Somalia**	**Nur et al., 2000**	No confirmation	BD + patients	EIA	None	1989	0% (0/256)	BD: 26 (18-40);<br>patients: (17-66);<br>children: (5months-9y)	BD 99%
Eastern Africa	**South Sudan**	**De thé et al., 1985**	No confirmation	NA	ELISA x2	None		9.2% (7/76)	(20-49)	NA
Eastern Africa	**Tanzania**	**De thé et al., 1985**	No confirmation	NA	ELISA x2	None		16.9% (79/468)	(0-50+)	NA
Eastern Africa	**Tanzania**	**De thé et al., 1989**	Other population (Patients)	Patients	ELISA	WB (DPN)	1985 +87	1.7% 0% (1+0/58+61)	NA	NA
Eastern Africa	**Tanzania**	**Schmutzhard et al., 1989**	Other population (Patients)	Patients	ELISA x2	None	1987	9.9% (25/253)	Med. 31 (1-78)	49.8%
Eastern Africa	**Tanzania**	**Howlett et al., 1990**	Other population (Patients)	Patients	ELISA	WB		0% (0/39)	(4-46)	77%
Eastern Africa	**Tanzania**	**Maselle et al., 1990**	No data	Asympto + patients	ELISA	WB		1% (NA/267)	NA	NA
Eastern Africa	**Tanzania**	**Matee et al., 1999**	No confirmation	BD	ELISA	None		12.7% (NA/300)	NA	NA
Eastern Africa	**Tanzania**	**Croce et al., 2007**	No confirmation	BD + PW	EIA	None	2002	2.1% (9 /430 PW);<br>3.7% (42/323 BD)	PW: 27.5 (15-50);<br>BD: 29.4 (16-60)	PW: 0%; BD:81.6%
Eastern Africa	**Uganda**	**Saxinger et al., 1984**	No confirmation	Pop + patients Burkitt	ELISA	NA		20.9% (18/86)		
Eastern Africa	**Uganda**	**De thé et al., 1985**	Other population (Patients)	Burkitt study	ELISA x2	None	1972-74	7.9% (8/101)	(10-50+)	NA
Eastern Africa	**Uganda**	**Larouze et al., 1985**	Other population (SW)	SW	RIPA			0.7% (1/135)	(20-40)	sex ratio M/F 1.04
Eastern Africa	**Uganda**	**Tabor et al., 1998**	Patients	Kaposi sarcoma	EIA	WB (2.4)	1969-70	12% (11/94)		
Eastern Africa	**Uganda**	**Biryahwaho et al., 1999**	No data (numbers NA)	PW	None	WB (2.4)		NA (NA/99)	NA	0%

Eastern Africa	**Uganda**	**Uchena Tweteise et al., 2015**	No confirmation	BD	ELISA x2	None	2014	0.5% (2/386)	Med. 18 (15-51)	62.2%
Eastern Africa	**Zambia**	**Tabor et al., 1990**		Rural pop +Patients	EIA	WB (NA) + RIPA	1976/81	0.4% (1/226 rural pop); 0% (0/123 patients)	NA	NA
Eastern Africa	**Zimbabwe**	**Emmanuel et al., 1989**		BD	ELISA + EIA	None	1987	0%(0/578 BD); 1.4% (4/296 HIV+); 0%(0/26 lymphoma patients)		
Eastern Africa	**Zimbabwe**	**Houston et al., 1994**		BD + patients	ELISA	IFA		0.1% (1/931 BD); 9.4% (3/32 neurological patients); 0% (88 HIV+; 23 multi-transfused; 8 haematological patients)	NA	NA
Southern Africa	**Eswatini/Swaziland**	**Van Rensburg et al., 1995**	Other population	Refugees				0% (NA/NA)		
Dependency of France	**Mayotte**	**Cnudde et al., 1991**	Other population (Patients)	Patients	ELISA	WB (DPN)	1988	0% (0/57 neurological and 140 internal medecine patients)	NA	NA
Southern Africa	**Namibia**	**Lecatsas et al., 1988**		Rural pop ?	ELISA	WB (DPN)		0% (0/407)	NA	NA
Southern Africa	**Namibia**	**Steele et al., 1994**	Other population (Children)	Children	ELISA x2	WB	1989	1% (3/289)	(5-19)	59%
Dependency of France	**Réunion**	**Schaffar-Deshayes et al., 1984**	No confirmation (& no data)	BD	RIPA	None		0% (NA/NA)	NA	NA
Dependency of France	**Réunion**	**Cnudde et al., 1991**		BD + patients	ELISA	WB (DPN)	1988	0% (0/258 BD); 1% (102 internal medecine patients + 136 neurological patients + 15 dialysis patients)	NA	NA
Dependency of France	**Réunion**	**Mahieux et al., 2009**		BD + patients	ELISA + PA + IFA	WB (2.2)	1988	0.03% (1/3,900 BD); 1.2% (3/257 patients)	BD: NA; Female patients: 44; Male patients: 50	BD: NA; Patients: 63%
Dependency of France	**Réunion**	**Aubry et al., 2013**		BD	ELISA	WB	2007-2012	0.01% (2/40,000 BD)	NA	NA
Dependency of France	**Réunion**	**Hoarau et al., 2017**	No data (numbers NA)	BD	ELISA	WB	2012-2016	0% (6/126,871 BD)		
Southern Africa	**Rep. of South Africa**	**Hunsmann et al., 1984**	Low numbers	Urban pop	ELISA			5% (1/20)	NA	NA
Southern Africa	**Rep. of South Africa**	**Saxinger et al., 1984**	No confirmation	BD + patients	ELISA	None	NA	5% (15/283 tumoral patients and BD)	NA	NA
Southern Africa	**Rep. of South Africa**	**Becker et al., 1985**	No data (numbers NA)	NA	ELISA		NA	0-4% (NA/543 black BD)	NA	NA
Southern Africa	**Rep. of South Africa**	**Neill et al., 1990**		BD	ELISA x2	WB	1990	0% (0/5603)		
Southern Africa	**Rep. of South Africa**	**Bhigjee et al., 1993**		Urban pop	ELISA x2	WB (DPN)		2.6% (26/1018)	31.5 +/- 12.7 (15-82)	48%
Southern Africa	**Rep. of South Africa**	**Goubau et al., 1993**		PW	ELISA	WB + IFA	NA	0.2% (1/428)	NA	0%
Southern Africa	**Rep. of South Africa**	**Taylor et al., 1992**	Other population	STDs	ELISA	None	1990	0% (0/492)	>16	49%
Southern Africa	**Rep. of South Africa**	**Taylor et al., 1996**		PW	PA	WB (2.3)	1993-94	0.6% (7/1,259)	NA	
Southern Africa	**Rep. of South Africa**	**Van der Ryst et al., 1996**		Rural + Urban pop + Patients	ELISA	WB		1.1% (178 rural pop); 2% (200 urban blacks); 0% (50 urban white pop); 33% (60 spastic paralysis patients); 0% (70 neurological patients); 0% (12 haematological malignancies), 6% (293 HIV)	NA	NA
Southern Africa	**Rep. of South Africa**	**du Plessis et al., 1999**	Patients	Bodies	NA	None		0% (NA/263)	NA	NA
Southern Africa	**Rep. of South Africa**	**Vandormael et al., 2017**	Other population (HIV patients)	HIV	NA			2.1% (8/388)		
Southern Africa	**Rep. of South Africa**	**Vermeulen et al., 2019**		BD	CLEIA	INNO-LIA + PCR	2013	0.13% (57/42,752 BD from 2013); 0.01% (3/37,422 BD from 1996)	(16-50+)	57.1%

n/N: number of HTLV-1 infected over the total number of people tested for each population (when available); * HTLV-1/2 seroprevalence; Rep.: Republic; NA: not available; Med. : median.

Abbreviated study population: BD: blood donors; CLL: chronic lymphatic leukaemia; DA: drug addicts; MSM: men having sex with men; NHL: Non-Hodgkin's lymphoma; PO: police officers; pop: population (rural or urban); PW: pregnant women; STI or STDs: sexual transmitted diseases patients; FSW: female sex workers; TB: tuberculosis patients.

Abbreviated screening and confirmatory assay: CLEIA: Chemiluminescent Immunoassay; IFA: immunofluorescence; INNO-LIA: Innogenetics line immunoassay;PA: particle agglutination assay; RIPA: immunoprecipitation; WB: Western blotting (DPN: DuPont de Nemours).
