# Supplementary material for: Geographic distribution, clinical epidemiology and genetic diversity of the human oncogenic retrovirus HTLV-1 in Africa, the world’s largest endemic area
Source: Front Immunol. 2023 Feb 3;14:1043600. doi: 10.3389/fimmu.2023.1043600 (PMC9935834; doi:10.3389/fimmu.2023.1043600)
Supplement: Supplementary file 2 [file Table_2.pdf]

Table S2: Epidemiological data and clinical status of 158 HTLV-1-infected individuals with Adult T-cell leukemia (ATL) and originating from 23 African countries and islands of the Indian Ocean

Region of origin	ID	Sex	Age (year)	Clinical status	Patient country origin	HTLV-1 genotype	Reference	Country of Research*
**North Africa**	BO	NA	NA	ATL	Algeria	NA	Gasmi M. et al., AIDS Res Hum Retrovir, 1994	France/Morocco
	KIN	F	44	ATL acute	Morocco	a-NA	Cassar O. et al., Emerg Mircrobes Infect, 2020	France
	MOR11	M	68	ATL acute	Morocco	NA	Rio B. et al., Presse Med, 1990	France
	AMC	F	33	ATL acute	Morocco	NA	Baas J. et al., Lancet, 1985	Netherlands
	PH507	M	25	ATL	Mauritania	a-NA	Desrames A. et al., J Virol, 2014	France
	MOH	M	23	ATL	Mauritania	NA	Michel P. et al., Med Trop, 1996	France
	MAU	M	53	ATL	Mauritania	NA	Gessain A. et al., Hum Retrovir HTLV, 1989	France
	COU	F	53	ATL acute	Mali	a-NA	Cassar O. et al., Emerg Mircrobes Infect, 2020	France
	KON.F	M	58	ATL chronic	Mali	a-NA	Cassar O. et al., Emerg Mircrobes Infect, 2020	France
	SIS	M	62	ATL acute	Mali	a-NA	Cassar O. et al., Emerg Mircrobes Infect, 2020	France
	KON.M	M	70	ATL lymphoma	Mali	a-WA	Cassar O. et al., Emerg Mircrobes Infect, 2020	France
	DIA.K	F	36	ATL acute	Mali	a-Sen	Cassar O. et al., Emerg Mircrobes Infect, 2020	France
	PH1248	F	65	ATL	Mali	a-WA	Desrames A. et al., J Virol, 2014	France
	MAL2	M	34	ATL smoldering	Mali	NA	Fouchard N. et al., Leukemia, 1998	France
	MAL3	M	38	ATL acute	Mali	NA	Fouchard N. et al., Leukemia, 1998	France
	PH71	M	45	ATL acute	Mali	a-NA	Mahieux R. et al., J Virol, 1997	France
	SEG	M	43	ATL	Mali	NA	Mahé A. et al., Ann Dermatol, 1994	Mali/France
**North-East Africa**	SUD6	M	57	ATL	Sudan	NA	Gorish B.M.T. et al., MedPress Oncology, 2019	Sudan
	EGY95	NA	[16-88]	ATL	Egypt	NA	Gill P.S. et al., N Engl J Med, 1995	United States
**West Africa**	COL	M	39	ATL smoldering	Senegal	a-Sen	Cassar O. et al., Emerg Mircrobes Infect, 2020	France
	THI	F	60	ATL chronic	Senegal	NA	Bentefouet T.L. et al., Ann Pathol, 2019	Senegal
	SEN6	F	60	ATL	Senegal	NA	Bentefouet T.L. et al., Ann Pathol, 2019	Senegal
	PH833	M	14	ATL	Senegal	a-WA	Desrames A. et al., J Virol, 2014	France
	PH961	M	54	ATL	Senegal	a-Sen	Desrames A. et al., J Virol, 2014	France
	PH1061	M	NA	ATL	Senegal	a-Sen	Desrames A. et al., J Virol, 2014	France
	HPD1	NA	NA	ATL acute	Senegal	NA	Gning S.B., Med Trop, 2006	Senegal
	HPD2	NA	NA	ATL acute	Senegal	NA	Gning S.B., Med Trop, 2006	Senegal
	HPD3	NA	NA	ATL acute	Senegal	NA	Gning S.B., Med Trop, 2006	Senegal
	HPD4	NA	NA	ATL acute	Senegal	NA	Gning S.B., Med Trop, 2006	Senegal
	HPD5	NA	NA	ATL lymphoma	Senegal	NA	Gning S.B., Med Trop, 2006	Senegal
	HPD6	NA	NA	ATL lymphoma	Senegal	NA	Gning S.B., Med Trop, 2006	Senegal
	PH821	F	17	ATL chronic	Senegal	NA	Mahé A. et al., Br J Dermatol, 2004	Senegal/France
	SEN3	F	43	ATL lymphoma	Senegal	NA	Gning S.B. et al., Dakar Med, 2003	Senegal
	SEN4	M	44	ATL lymphoma	Senegal	NA	Gning S.B. et al., Dakar Med, 2003	Senegal
	SEN5	M	42	ATL	Senegal	NA	Poszepczynska-Guigné E. et al., Ann Derm Venerol, 2006	Senegal/France
	TDG	F	41	ATL acute	Senegal	NA	Mbaye P.S. et al., Dakar Med, 1998	Senegal
	OH	M	23	ATL acute	Senegal	NA	Mbaye P.S. et al., Dakar Med, 1998	Senegal
	HUDF1	F	50	ATL lymphoma	Senegal	NA	Michel P. et al., Med Trop, 1996	Senegal/France
	HUDF2	F	41	ATL	Senegal	NA	Michel P. et al., Med Trop, 1996	Senegal/France
	NYC	NA	NA	ATL	The Gambia	NA	Philips A.A. et al., Cancer, 2010	United States
	SL3	M	29	ATL	Sierra Leone	NA	Stewart J.S.W. et al., Lancet, 1984	United Kingdom
	BAL4	M	41	ATL chronic	Guinea	a-NA	Cassar O. et al., Emerg Mircrobes Infect, 2020	France
	PH541	F	21	ATL	Guinea	a-G-Rec	Cassar O. et al., Emerg Mircrobes Infect, 2020	France
	CON.B	F	32	ATL lymphoma	Guinea	a-WA	Cassar O. et al., Emerg Mircrobes Infect, 2020	France
	LIB95	NA	[16-88]	ATL	Liberia	NA	Gill P.S. et al., N Engl J Med, 1995	United States
	PH482	M	53	ATL	Guinea-Bissau	a	Mahieux R. et al., J Virol, 1997	France
	KOU.Y	M	41	ATL lymphoma	Côte d'Ivoire	a-WA	Cassar O. et al., Emerg Mircrobes Infect, 2020	France
	IC1682	M	37	ATL	Côte d'Ivoire	a-WA	Cassar O. et al., Emerg Mircrobes Infect, 2020	France
	DAF	M	62	ATL acute	Côte d'Ivoire	a-WA	Cassar O. et al., Emerg Mircrobes Infect, 2020	France
	GBA	M	51	ATL chronic	Côte d'Ivoire	a-WA	Cassar O. et al., Emerg Mircrobes Infect, 2020	France
	GNA6	F	57	ATL chronic	Côte d'Ivoire	a-WA	Cassar O. et al., Emerg Mircrobes Infect, 2020	France
	GNA7	M	51	ATL lymphoma	Côte d'Ivoire	a-WA	Cassar O. et al., Emerg Mircrobes Infect, 2020	France
	KEI	F	33	ATL lymphoma	Côte d'Ivoire	a-NA	Cassar O. et al., Emerg Mircrobes Infect, 2020	France
	NGO	F	42	ATL chronic	Côte d'Ivoire	a-WA	Cassar O. et al., Emerg Mircrobes Infect, 2020	France
	SAK	M	39	ATL smoldering	Côte d'Ivoire	a-WA	Cassar O. et al., Emerg Mircrobes Infect, 2020	France
	DRE	F	43	ATL acute	Côte d'Ivoire	a-WA	Cassar O. et al., Emerg Mircrobes Infect, 2020	France
	PH610	M	59	ATL	Côte d'Ivoire	a-WA	Desrames A. et al., J Virol, 2014	France
	PH681	M	35	ATL	Côte d'Ivoire	a-WA	Desrames A. et al., J Virol, 2014	France
	PH1359	F	54	ATL	Côte d'Ivoire	a-Sen	Desrames A. et al., J Virol, 2014	France
	PH52	M	NA	ATL	Côte d'Ivoire	a-WA	Mahieux R. et al., J Virol, 1997	France
	SLH12	F	36	ATL	Côte d'Ivoire	NA	Gessain A. et al., Cancer, 1992	France
	COI2	M	42	ATL acute	Côte d'Ivoire	NA	Rio B. et al., Presse Med, 1990	France
	COI6	F	49	ATL acute	Côte d'Ivoire	NA	Rio B. et al., Presse Med, 1990	France
	COI7	F	19	ATL acute	Côte d'Ivoire	NA	Rio B. et al., Presse Med, 1990	France
	COI8	M	21	ATL acute	Côte d'Ivoire	NA	Rio B. et al., Presse Med, 1990	France
	COI12	F	45	ATL	Côte d'Ivoire	NA	Gessain A. et al., Blood, 1990	France
	UHA◆	F	19	ATL	Côte d'Ivoire	NA	Baurmann H. et al., Am J Med, 1988	France
	KOA.H	F	35	ATL acute	Burkina Faso	a-WA	Cassar O. et al., Emerg Mircrobes Infect, 2020	France
	KWA	M	61	ATL chronic	Ghana	a-WA	Cassar O. et al., Emerg Mircrobes Infect, 2020	France
	GHA1	F	48	ATL chronic	Ghana	NA	Lahoud O.B. et al., Bone Marrow Transplant, 2018	United States
	GHA2	F	33	ATL chronic	Ghana	NA	Edwards C.M.B. et al., J R Soc Med, 2003	United Kingdom
	MOG.B	F	56	ATL lymphoma	Togo	a-WA	Cassar O. et al., Emerg Mircrobes Infect, 2020	France
	AHO.M	M	56	ATL acute	Togo	b	Cassar O. et al., Emerg Mircrobes Infect, 2020	France

Region of origin	ID	Sex	Age (year)	Clinical status	Patient country origin	HTLV-1 genotype	Reference	Country of Research*
	NIG2	M	32	ATL	Nigeria	NA	Avallone G. et al., Am J Trop Med Hyg, 2021	Italy
	NIG1	M	32	ATL	Nigeria	NA	Boodman C. et al., Open Forum Infect Dis, 2020	Canada
	IYA	M	42	ATL acute	Nigeria	b	Cassar O. et al., Emerg Mircrobes Infect, 2020	France
	DES.P	M	28	ATL lymphoma	Nigeria	a-TC	Cassar O. et al., Emerg Mircrobes Infect, 2020	France
	NIG.IT	F	27	ATL acute	Nigeria	NA	Re, M.C. et al., New Microbiol, 2004	Italy
	K0250	M	47	ATL	Nigeria	NA	Williams C.K.O. et al., Br J Cancer, 1993	Nigeria/United States
	K0319	M	12	ATL	Nigeria	NA	Williams C.K.O. et al., Br J Cancer, 1993	Nigeria/United States
	K1282	F	39	ATL	Nigeria	NA	Williams C.K.O. et al., Br J Cancer, 1993	Nigeria/United States
	K4950	F	22	ATL	Nigeria	NA	Williams C.K.O. et al., Br J Cancer, 1993	Nigeria/United States
	NIG.M	M	60	ATL	Nigeria	NA	Fleming A.F. et al., Int J Cancer, 1986	Nigeria/Germany
	NIG4	M	19	ATL	Nigeria	NA	Williams C.K.O. et al., Br Med J, 1984	Nigeria/United States
	NIG8	F	57	ATL	Nigeria	NA	Williams C.K.O. et al., Br Med J, 1984	Nigeria/United States
**Central Africa**	EL	M	30	ATL	DRC (ex-Zaïre)	b	Hahn B. et al., Int J Cancer, 1984	United States
	GAB5	F	58	ATL	Gabon	NA	Perret J-L. et al., Med Trop, 1996	Gabon/France
	GAB1	F	35	ATL	Gabon	NA	Delaporte E. et al., Int J Cancer, 1993	Gabon/France
	GAB2	F	48	ATL	Gabon	NA	Delaporte E. et al., Int J Cancer, 1993	Gabon/France
	GAB3	F	52	ATL	Gabon	NA	Delaporte E. et al., Int J Cancer, 1993	Gabon/France
	GAB4	H	42	ATL	Gabon	NA	Delaporte E. et al., Int J Cancer, 1993	Gabon/France
	GAB14	M	27	ATL	Gabon	NA	Rio B. et al., Presse Med, 1990	France
**Southern Africa**	PH549	M	32	ATL	Angola	a-TC	Desrames A. et al., J Virol., 2014	France
	AFS1	M	33	ATL	RSA	NA	John TJ. et al., QJM, 2020,	South Africa
	AFS2◆	F	37	ATL	RSA	NA	Laher AE. et al., Oxf Med Case Reports, 2018	South Africa
	AFS3	F	49	ATL	RSA	NA	Patel M. et al., South Afr J Epidemiol Infect, 1995	South Africa
	AFSNat1	M	66	ATL	RSA	NA	Jogessar V.B. et al., S Afr Med J, 1992	South Africa
	AFSNat2	F	28	ATL	RSA	NA	Jogessar V.B. et al., S Afr Med J, 1992	South Africa
**Indian Ocean**	CHU3	F	56	ATL	The Reunion Island	NA	Hoarau G. et al., Med Mal Infect, 2017	France
	CHU4	M	54	ATL lymphoma	The Reunion Island	NA	Hoarau G. et al., Med Mal Infect, 2017	France
	SEY2	M	25	ATL lymphoma	Republic of Seychelles	NA	Mistro A. et al., Leukemia and Lymphoma, 1992	Italy
**African region and/or aggregated cases**								
**North Africa**	MAR	M	60	ATL	NA	NA	Tubiana N. et al., Lancet, 1985	France
**West Africa**	WAFR	M	58	ATL	NA	NA	Gorelik N. et al., Skeletal Radiol, 2020	United States
	8 cases	5F/3M	[22-78]	ATL	Senegal	NA	Dioussé P., Rev Afr Malg Rech Sc, 2017	Senegal
**Northwestern Africa**	NWAFR	M	53	ATL acute	NA	NA	Abdullgaffar B. and Abdulrahman S., J Cutan Pathol, 2021	United Arab Emirates
**Southern Africa**	31 cases	16F/15M	[17-80]	ATL acute	RSA	NA	Nell E.M et al., J Cancer Epidemiol, 2022	South Africa
**Africa**	5 cases	NA	NA	ATL	NA	NA	De Mendoza C. et al., Open Forum Infect Dis, 2019	Spain
	10 cases	NA	NA	ATL	NA	NA	Ireland G. et al., Euro Surveill, 2017	United Kingdom

* Based on the affiliation of the three first and last authors

◆ HIV co-infected patient

ATL: Adult T-cell Leukemia; NA: Not available

a-NA, a-WA, a-Sen, a-TC: North African, West African, Senegalese and Transcontinental clades of the HTLV-1a gentotype

a-G-rec: Recombinant strain from Guinea and Ghana of the HTLV-1a genotype

DRC: Democratic Republic of the Congo, RSA: Republic of South Africa
