# Supplementary material for: Geographic distribution, clinical epidemiology and genetic diversity of the human oncogenic retrovirus HTLV-1 in Africa, the world’s largest endemic area
Source: Front Immunol. 2023 Feb 3;14:1043600. doi: 10.3389/fimmu.2023.1043600 (PMC9935834; doi:10.3389/fimmu.2023.1043600)
Supplement: Supplementary file 3 [file Table_3.pdf]

Table S3: Epidemiological data and clinical status of 361 HTLV-1-infected individuals with Tropical spastic paraparesis or HTLV-1 associated myelopathy (TSP/HAM) and originating from 25 African countries and islands of the Indian Ocean

Region of origin	ID	Sex	Age (year)	Patient country origin	HTLV-1 genotype	Reference	Country of research origin*
**North Africa**	Mau1371	M	51	Mauritania	a-Sen	Cassar O. et al., Emerg Mircrobes Infect, 2020	France
	OD	NA	NA	Mauritania	a-NA	Gasmi M. et al., AIDS Res Hum Retrovir, 1994	Morocco/France
	PH1620	F	32	Mali	a-NA	Cassar O. et al., Emerg Mircrobes Infect, 2020	France
	CHIR1	F	55	Morocco	NA	Boucetta M. et al., Neurologie tropicale, 1993	Morocco
	CHIR2	F	34	Morocco	NA	Boucetta M. et al., Neurologie tropicale, 1993	Morocco
	CHIR3	F	26	Morocco	NA	Boucetta M. et al., Neurologie tropicale, 1993	Morocco
	PR52	F	33	Morocco	a-NA	Farouqi B. et al., AIDS Res Hum Retrovir, 1992	Morocco
	PR144	F	50	Morocco	a-NA	Farouqi B. et al., AIDS Res Hum Retrovir, 1992	Morocco
	PC8	F	31	Morocco	NA	Farouqi B. et al., AIDS Res Hum Retrovir, 1992	Morocco
	P1041	M	29	Morocco	NA	Farouqi B. et al., AIDS Res Hum Retrovir, 1992	Morocco
**North-East Africa**	EGY1	M	24	Egypt	NA	Hassan A. et al., J Neurovirol, 1996	Egypt/Japan
	EGY2	M	26	Egypt	NA	Hassan A. et al., J Neurovirol, 1996	Egypt/Japan
**West Africa**	PH1635	M	79	Senegal	a-Sen	Cassar O. et al., Emerg Mircrobes Infect, 2020	France
	PH578	M	62	Senegal	a-TC	Desrames A. et al., J Virol, 2014	France
	PH121	F	47	Senegal	a-TC	Mahieux R. et al., J Virol, 1997	France
	PH122	F	60	Senegal	a-WA	Mahieux R. et al., J Virol, 1997	France
	DAK1	M	60	Senegal	NA	Michel P. et al., Med Trop, 1996	France/Senegal
	DAK2	F	47	Senegal	NA	Michel P. et al., Med Trop, 1996	France/Senegal
	DAK3	M	34	Senegal	NA	Michel P. et al., Med Trop, 1996	France/Senegal
	DAK4	F	40	Senegal	NA	Michel P. et al., Med Trop, 1996	France/Senegal
	DAK5	M	58	Senegal	NA	Michel P. et al., Med Trop, 1996	France/Senegal
	DAK6◆	F	52	Senegal	NA	Michel P. et al., Med Trop, 1996	France/Senegal
	FAN1◆	F	37	Senegal	NA	Diop A. G. et al., Neurol Trop, 1993	Senegal
	FAN2◆	F	40	Senegal	NA	Diop A. G. et al., Neurol Trop, 1993	Senegal
	FAN3◆	M	55	Senegal	NA	Diop A. G. et al., Neurol Trop, 1993	Senegal
	SEN1	NA	NA	Senegal	NA	Gessain A. et al., Cancer Res, 1990	France
	SEN20	M	44	Senegal	NA	Gout O. et al., Arch Neurol, 1989	France
	SEN19	M	43	Senegal	NA	Gessain A. et al., J Infect Dis, 1988	France
	GUI1	NA	9	Guinea	NA	Quintas S. et al., Rev Neurol, 2004	Portugal
	NIH00261	M	65	Liberia	NA	Enose-Akahata Y. et al., Retrovir, 2016	United States
	N26	M	74	Ghana	a-WA	Desrames A. et al., J Virol, 2014	France
	DIA.H	F	40	Côte d'Ivoire	a-Sen	Cassar O. et al., Emerg Mircrobes Infect, 2020	France
	PH111◆	M	40	Côte d'Ivoire	a-WA	Desrames A. et al., J Virol, 2014	France
	TBX	M	35	Côte d'Ivoire	a-WA	Desrames A. et al., J Virol, 2014	France/United Kingdom
	CI-Akr	M	45	Côte d'Ivoire	NA	Komurian F et al., J Virol., 1991	France
	Patient 16	NA	NA	Côte d'Ivoire	NA	Hugon J. et al., J Neurol Neurosurg Psychiatry, 1990	France/Côte d'Ivoire
	Patient 17	NA	NA	Côte d'Ivoire	NA	Hugon J. et al., J Neurol Neurosurg Psychiatry, 1990	France/Côte d'Ivoire
	Patient 18	NA	NA	Côte d'Ivoire	NA	Hugon J. et al., J Neurol Neurosurg Psychiatry, 1990	France/Côte d'Ivoire
	CI57	M	26	Côte d'Ivoire	NA	Giordano C. et al., Rev Neurol, 1988	Côte d'Ivoire/France
	CI17	M	32	Côte d'Ivoire	NA	Gessain A. et al., J Infect Dis, 1988	France
	CI18	M	33	Côte d'Ivoire	NA	Gessain A. et al., J Infect Dis, 1988	France
	CI1◆	M	32	Côte d'Ivoire	NA	Gessain A. et al., Lancet, 1986	France
	NIG	F	40	Niger	NA	Develoux M et al., Med Trop, 1996	Senegal/Niger/France
	Nig21	F	51	Nigeria	a-TC	Cassar O. et al., Emerg Mircrobes Infect, 2020	France
	Nig64	F	72	Nigeria	a-WA	Cassar O. et al., Emerg Mircrobes Infect, 2020	France
	TBU	F	66	Nigeria	b	Desrames A. et al., J Virol, 2014	France
**Central Africa**	GAB3	M	>60	Gabon	b	Mahieux R. et al., J Virol, 1997	France
	GAB4	M	>65	Gabon	b	Mahieux R. et al., J Virol, 1997	France
	PH236	F	70	Gabon	b	Mahieux R. et al., J Virol, 1997	France
	SDen	M	NA	Gabon	b	Mahieux R. et al., J Virol, 1997	France
	GAB5	M	66	Gabon	a-NA	Perret J.L. et al., 1990	Gabon
	GAB7	M	77	Gabon	b	Perret J.L. et al., 1990	Gabon
	GAB1	M	55	Gabon	a-NA	Delaporte E. et al., Lancet, 1989	Gabon/Belgium
	GAB2	M	60	Gabon	b	Delaporte E. et al., Lancet, 1989	Gabon/Belgium
	PH1319	F	50	Chad	a-TC	Desrames A. et al., J Virol, 2014	France
	PH179	F	38	Chad	a-NA	Mahieux R. et al., J Virol, 1997	France
	PH63	M	34	Republic of the Congo	a-TC	Desrames A. et al., J Virol, 2014	France
	PH511	F	46	CAR	b	Hervé V. et al., Presse Médicale, 1994	CAR/France
	CAR9	F	34	CAR	a-NA	Gessain A. et al., J Gen Virol, 1990	France
	CAR1	NA	NA	CAR	a-NA	Gessain A. et al., Cancer Res, 1990	France
	PH648	F	57	DRC (ex-Zaire)	a-NA	Desrames A. et al., J Virol, 2014	France
	PH1225	M	54	DRC (ex-Zaire)	a-TC	Desrames A. et al., J Virol, 2014	France
	ZAI3	M	35	DRC (ex-Zaire)	NA	Ossemann M. et al., Acta Neurol Belg, 1996	Belgium
	ZAI4	F	62	DRC (ex-Zaire)	NA	Ossemann M. et al., Acta Neurol Belg, 1996	Belgium
	PH198	F	54	DRC (ex-Zaire)	a-NA	Mahieux R. et al., J Virol, 1997	France
	K435	M	16	DRC (ex-Zaire)	b	Mahieux R. et al., J Virol, 1997	France
	MAS	F	56	DRC (ex-Zaire)	b	Mahieux R. et al., J Virol, 1997	France
	ME	F	30	DRC (ex-Zaire)	a-NA	de Saussure P. et al., Schweiz Med Wochenschr, 1991	Switzerland
	BL	F	42	DRC (ex-Zaire)	a-NA	de Saussure P. et al., Schweiz Med Wochenschr, 1991	Switzerland
	ZAI7	F	56	DRC (ex-Zaire)	a-NA	Gessain A. et al., J Gen Virol, 1990	France
	ZAI1	NA	NA	DRC (ex-Zaire)	a-NA	Gessain A. et al., Cancer Res, 1990	France
	ZAIB	M	32	DRC (ex-Zaire)	NA	Taelman H. et al., Ann Soc Belge Med Trop, 1989	Belgium

Region of origin	ID	Sex	Age (year)	Patient country origin	HTLV-1 genotype	Reference	Country of research origin*
**East Africa**	ETH1	M	38	Ethiopia	NA	Abebe M. et al., Trans R Soc Trop Med Hyg., 1991	Ethiopia/Sweden/France
	ETH2	NA	NA	Ethiopia	NA	Abebe M. et al., Trans R Soc Trop Med Hyg., 1991	Ethiopia/Sweden/France
	ETH3	M	31	Ethiopia	NA	Ryberg B. et al., Br Med J, 1987	Sweden
	ETH4	F	45	Ethiopia	NA	Dereje M. et al., Ethiop J Health Sci, 2022	Ethiopia
**Southern Africa**	TCY	F	52	Eswatini (ex-Swaziland	a-TC	Desrames A. et al., J Virol, 2014	France/United Kingdom
	TDS	F	62	Zambia	b	Desrames A. et al., J Virol, 2014	France/United Kingdom
	3TDJ	F	55	Zimbabwe	a-TC	Desrames A. et al., J Virol, 2014	France/United Kingdom
	Zim1◆	M	53	Zimbabwe	NA	Houston S. et al., Trans R Soc Trop Med Hyg., 1994	Canada/Zimbabwe
	Zim2◆	F	21	Zimbabwe	NA	Houston S. et al., Trans R Soc Trop Med Hyg., 1994	Canada/Zimbabwe
	Zim3◆	F	38	Zimbabwe	NA	Houston S. et al., Trans R Soc Trop Med Hyg., 1994	Canada/Zimbabwe
	97MZ03	NA	NA	Mozambique	a	Engelbrecht S. et al., AIDS Res Hum Retroviruses, 1999	South Africa/Mozambique
	97MZ06	NA	NA	Mozambique	a	Engelbrecht S. et al., AIDS Res Hum Retroviruses, 1999	South Africa/Mozambique
	97MZ26	NA	NA	Mozambique	a	Engelbrecht S. et al., AIDS Res Hum Retroviruses, 1999	South Africa/Mozambique
	97MZ28	NA	NA	Mozambique	a	Engelbrecht S. et al., AIDS Res Hum Retroviruses, 1999	South Africa/Mozambique
	SANBS480	M	65	RSA	a-TC	Vermeulen M. et al., Vox Sang, 2019	France/South Africa
	afs5	M	48	RSA	NA	Schutte C-M. et al., S Afr Med J, 2013	South Africa
	afs6	M	47	RSA	NA	Schutte C-M. et al., S Afr Med J, 2013	South Africa
	afs7	M	44	RSA	NA	Schutte C-M. et al., S Afr Med J, 2013	South Africa
	afs8	M	43	RSA	NA	Schutte C-M. et al., S Afr Med J, 2013	South Africa
	afs9	F	21	RSA	NA	Schutte C-M. et al., S Afr Med J, 2013	South Africa
	afs10	M	47	RSA	NA	Schutte C-M. et al., S Afr Med J, 2013	South Africa
	afs11	F	43	RSA	NA	Schutte C-M. et al., S Afr Med J, 2013	South Africa
	afs12	M	51	RSA	NA	Schutte C-M. et al., S Afr Med J, 2013	South Africa
	afs13	M	57	RSA	NA	Schutte C-M. et al., S Afr Med J, 2013	South Africa
	afs14	M	41	RSA	NA	Schutte C-M. et al., S Afr Med J, 2013	South Africa
	afs15	F	39	RSA	NA	Schutte C-M. et al., S Afr Med J, 2013	South Africa
	afs16◆	M	34	RSA	NA	Schutte C-M. et al., S Afr Med J, 2013	South Africa
	afs17◆	M	42	RSA	NA	Schutte C-M. et al., S Afr Med J, 2013	South Africa
	afs18◆	M	32	RSA	NA	Schutte C-M. et al., S Afr Med J, 2013	South Africa
	afs19◆	F	36	RSA	NA	Schutte C-M. et al., S Afr Med J, 2013	South Africa
	afs20◆	F	25	RSA	NA	Schutte C-M. et al., S Afr Med J, 2013	South Africa
	afs16◆	M	36	RSA	NA	Schutte C-M. et al., S Afr Med J, 2013	South Africa
	afs16◆	F	45	RSA	NA	Schutte C-M. et al., S Afr Med J, 2013	South Africa
	afs16◆	F	31	RSA	NA	Schutte C-M. et al., S Afr Med J, 2013	South Africa
	afs16◆	F	49	RSA	NA	Schutte C-M. et al., S Afr Med J, 2013	South Africa
	TBH98	NA	NA	RSA	a	Engelbrecht S. et al., AIDS Res Hum Retroviruses, 1999	South Africa/Mozambique
	afs911	F	45	RSA	a-TC	Mahieux R. et al., J Virol, 1997	France
	afs1	M	61	RSA	a-TC	Mahieux R. et al., J Virol, 1997	France
	afs2	M	40	RSA	a-TC	Mahieux R. et al., J Virol, 1997	France
	afs3	F	44	RSA	a-TC	Mahieux R. et al., J Virol, 1997	France
	afsgaz	M	33	RSA	NA	Joubert J. et al., S Afr Med J, 1991	South Africa
	afs4	M	49	RSA	NA	Bhigjee A.I. et al., S Afr Med J, 1989	South Africa
**Indian Ocean**	PH1361	F	52	Comoros	a-TC	Desrames A. et al., J Virol, 2014	France
	CHU1	F	76	The Reunion Island	NA	Hoarau G. et al., Med Mal Infect, 2017	France
	CHU2	F	60	The Reunion Island	NA	Hoarau G. et al., Med Mal Infect, 2017	France
	VidD	F	59	The Reunion Island	a	Mahieux R. et al., AIDS Res Hum Retroviruses, 1994	France
	REU1	M	77	The Reunion Island	NA	Cnudde F. et al., JAIDS, 1991	France
	SEY1	F	64	Republic of Seychelles	NA	Thyagarajan D. et al., Med J Aust, 1993	Australia
	JB	M	27	Republic of Seychelles	NA	Roman G.C. et al., Arch Neurol, 1987	United States
	AL	F	38	Republic of Seychelles	NA	Roman G.C. et al., Arch Neurol, 1987	United States
	ED	F	31	Republic of Seychelles	NA	Roman G.C. et al., Arch Neurol, 1987	United States
	CC	M	50	Republic of Seychelles	NA	Roman G.C. et al., Arch Neurol, 1987	United States
	MF	F	52	Republic of Seychelles	NA	Roman G.C. et al., Arch Neurol, 1987	United States
	AB	F	58	Republic of Seychelles	NA	Roman G.C. et al., Arch Neurol, 1987	United States
	AD	F	59	Republic of Seychelles	NA	Roman G.C. et al., Arch Neurol, 1987	United States
	YJ	F	41	Republic of Seychelles	NA	Roman G.C. et al., Arch Neurol, 1987	United States
	WG	M	64	Republic of Seychelles	NA	Roman G.C. et al., Arch Neurol, 1987	United States
	YH	F	59	Republic of Seychelles	NA	Roman G.C. et al., Arch Neurol, 1987	United States
	RJ	F	33	Republic of Seychelles	NA	Roman G.C. et al., Arch Neurol, 1987	United States
	SR	M	66	Republic of Seychelles	NA	Roman G.C. et al., Arch Neurol, 1987	United States
	EL	F	53	Republic of Seychelles	NA	Roman G.C. et al., Arch Neurol, 1987	United States
	PF	F	52	Republic of Seychelles	NA	Roman G.C. et al., Arch Neurol, 1987	United States
**Aggregated cases**							
**West Africa**	9 cases	NA	15-74	Togo	NA	Balogou A.A.K. et al., Bull Soc Path Exot, 2000	Togo/France
**Central Africa**	10 cases	NA	NA	DRC (ex-Zaire)	NA	Liu H.F., et al., AIDS Res Hum Retroviruses, 1994	Belgium
	25 cases	16F/9M	NA	DRC (ex-Zaire)	NA	Kayembe K. et al., J Neurol Neurosurg Psychiatry,1990	DRC/Belgium
	9 cases		1-69	DRC (ex-Zaire)	NA	Kazadi K. et al., The Lancet, 1990	DRC/France
**South Africa**	20 cases	NA	NA	RSA	NA	Van der Ryst E. et al., Centr Afr J Med, 1996	South Africa
	90 cases	NA	NA	RSA	NA	Bhigjee A.I. et al., S Afr Med J, 1993	South Africa
	40 cases	NA	NA	RSA	NA	Bhigjee A.I. et al., J Neurol Neurosurg Psychiatry, 1992	South Africa
	24 cases	17F/7M	20-70	RSA	NA	Bhigjee A.I. et al., Brain, 1990	South Africa

* Based on the affiliation of the three first and last authors
◆ HIV co-infected patient
NA: Not available
a-NA, a-WA, a-Sen, a-TC: North African, West African, Senegalese and Transcontinental clades of the HTLV-1a gentotype
CAR: Central African Republic, DRC: Democratic Republic of the Congo, RSA: Republic of South Africa
